# Horizontal Gene Transfer and Tandem Duplication Shape the Unique CAZyme Complement of the Mycoparasitic Oomycetes *Pythium oligandrum* and *Pythium periplocum*

**DOI:** 10.3389/fmicb.2020.581698

**Published:** 2020-10-29

**Authors:** Dong Liang, Christian Benjamin Andersen, Ramesh R. Vetukuri, Daolong Dou, Laura J. Grenville-Briggs

**Affiliations:** ^1^College of Plant Protection, Nanjing Agricultural University, Nanjing, China; ^2^Department of Plant Protection Biology, Swedish University of Agricultural Sciences, Alnarp, Sweden

**Keywords:** mycoparasitism, CAZy, carbohydrate active enzymes, cell wall degrading enzymes, biological control, comparative genomics, oomycete genomics

## Abstract

Crop protection strategies that are effective but that reduce our reliance on chemical pesticides are urgently needed to meet the UN sustainable development goals for global food security. Mycoparasitic oomycetes such *as Pythium* oligandrum and *Pythium periplocum*, have potential for the biological control of plant diseases that threaten crops and have attracted much attention due to their abilities to antagonize plant pathogens and modulate plant immunity. Studies of the molecular and genetic determinants of mycoparasitism in these species have been less well developed than those of their fungal counterparts. Carbohydrate-active enzymes (CAZymes) from *P. oligandrum* and *P. periplocum* are predicted to be important components of mycoparasitism, being involved in the degradation of the cell wall of their oomycete and fungal prey species. To explore the evolution of CAZymes of these species we performed an *in silico* identification and comparison of the full CAZyme complement (CAZyome) of the two mycoparasitic *Pythium* species (*P. oligandrum* and *P. periplocum*), with seven other *Pythium* species, and four *Phytophthora* species. Twenty CAZy gene families involved in the degradation of cellulose, hemicellulose, glucan, and chitin were expanded in, or unique to, mycoparasitic *Pythium* species and several of these genes were expressed during mycoparasitic interactions with either oomycete or fungal prey, as revealed by RNA sequencing and quantitative qRT-PCR. Genes from three of the cellulose and chitin degrading CAZy families (namely AA9, GH5_14, and GH19) were expanded via tandem duplication and predominantly located in gene sparse regions of the genome, suggesting these enzymes are putative pathogenicity factors able to undergo rapid evolution. In addition, five of the CAZy gene families were likely to have been obtained from other microbes by horizontal gene transfer events. The mycoparasitic species are able to utilize complex carbohydrates present in fungal cell walls, namely chitin and N-acetylglucosamine for growth, in contrast to their phytopathogenic counterparts. Nonetheless, a preference for the utilization of simple sugars for growth appears to be a common trait within the oomycete lineage.

## Introduction

The oomycetes are notorious as plant pathogens that cause devastating diseases in crop plants and our natural landscapes. Efficient control of many oomycete diseases relies on the usage of synthetic pesticides, which may have detrimental effects on the environment. The Genus *Pythium* contains predominantly saprotrophs and necrotrophic pathogens that occupy diverse ecological niches and infect various plant, arthropod, and even, human hosts (Plaats-Niterink, 1981). Some members of this fungal-like eukaryotic lineage are mycoparasites, obtaining nutrients from living fungal or oomycete hosts and are thus of great interest as potential biological control agents as part of an Integrated Pest Management (IPM) system for the control of crop diseases.

Mycoparasitic oomycetes have been less-well studied than their plant pathogenic counterparts, nevertheless, two species, *Pythium oligandrum* and *Pythium periplocum* have been investigated in this context. *P. oligandrum* has been documented as an antagonist and/or mycoparasite of a wide range of hosts including plant pathogenic *Pythium* spp. (Benhamou and Chet, 1997), *Phytophthora* spp. (Picard et al., 2000a; Horner et al., 2012), ascomycetes and basidiomycetes (Bradshaw-Smith et al., 1991; Benhamou et al., 1997). As well as an ability to antagonize fungi and oomycetes, these species display several other key features important for biological control of plant diseases. They typically grow faster than their plant pathogenic counterparts, meaning that they can outcompete other species for rhizosphere space and nutrition and they are able to promote both plant growth (Le Floch et al., 2003), and induced resistance in host plants (Takenaka et al., 2011; Ouyang et al., 2015). Whilst these experiments demonstrate their potential as biological control agents and detailed microscopic analysis has revealed the nature of their mycoparasitic interactions with various prey species (Benhamou et al., 1999; Picard et al., 2000b), there have been fewer mechanistic studies investigating the molecular or genetic determinants of their mycoparasitic lifestyle. Several cell wall-degrading enzymes and putative effectors were previously revealed to be expressed by *P. oligandrum* in the presence of oomycete tissue (Horner et al., 2012) and microarray analysis was recently used to investigate *P. oligandrum-*plant interactions (Yacoub et al., 2018), which showed significant reprogramming of the *Vitus virifera* root transcriptome in the presence of *P. oligandrum.* However, these studies were limited either in their methodology (a small sequencing study of 3,000 cDNA clones) or scope (focus on changes in plant roots), respectively, and thus we still lack a detailed mechanistic understanding of mycoparasitism in the oomycete lineage.

To provide a more complete basis for detailed molecular and genetic analysis of mycoparasitic *Pythium* species, we have sequenced and assembled the genomes of both *P. oligandrum* (Kushwaha et al., 2017a) and *P. periplocum* (Kushwaha et al., 2017b). Two other isolates of *P. oligandrum* have also been sequenced (Berger et al., 2016; Faure et al., 2020). We have also performed RNA sequencing of selected *Pythium oligandrum-*prey and *Pythium periplocum*-prey interactions, a detailed analysis of which will be published elsewhere.

The Carbohydrate-active enzyme complement (CAZyome) is the repertoire of predicted genes coding for enzymes involved in carbohydrate metabolism in an organism (CAZymes), including the synthesis, degradation, and modification of structural components of the cell wall. The CAZymes can be divided into five superfamilies, glycoside hydrolases (GH), glycosyl transferases (GT), polysaccharide lyases (PL), and carbohydrate esterases (CE) based on their activity and sequence similarity (Lombard et al., 2013). Comparative analysis of the mycoparasitic fungi *Trichoderma atrovidirde* and *Trichoderma virens* with other closely related fungal species, reveals the expansion of the CAZyome, particularly of genes from the family GH18, which comprises proteins with putative functions as chitinases in the mycoparasitic *Trichoderma* species. Other CAZy families expanded were those encoding endo-β-N acetylglucosimindases and β-1,3-glucanases from the families GH17, GH55, GH64, and GH81 (Kubicek et al., 2011). Chitin is a major component of the fungal cell wall, and therefore an obvious target for mycoparasitic lytic attack. Carbohydrate binding domains (CBMs) are also more abundant in the chitinase sequences from mycoparasitic *Trichoderma* species, compared to other fungi. As well as an expansion in the number of genes encoding chitinases, these *Trichoderma* genomes also contain an expanded number of GH75 chitosanases. It has long been known that the binding and degradation of chitin is important for successful mycoparasitism in these *Trichoderma* species. *T. virens* strains with chitinase knock-out mutants show reduced mycoparasitic ability, whilst strains that constitutively overexpress the same gene show enhanced biocontrol capabilities (Baek et al., 1999). The addition of CBMs to chitinases from *Trichoderma harzianum* has been shown to increase the antifungal activity of this species (Limón et al., 2004). Interestingly, Druzhinina et al. (2018) found nearly half of CAZy families in the mycoparasitic *Trichoderma* species were obtained by lateral gene transfer from plant-associated filamentous Ascomycete fungi, which has allowed *Trichoderma* species to expand their nutritional base (Druzhinina et al., 2018).

In the present study, we report the detailed mining of the *P. oligandrum* and *P. periplocum* genomes to investigate the presence and role of genes encoding CAZymes. Expression was investigated through analysis of RNA sequencing data from *Pythium oligandrum-Phytopthora infestans* and *Pythium periplocum-Ph. infestans* as well as *P. periplocum-Botryis cinerea* interactions. Transcript abundance of selected genes was confirmed through qRT-PCR analysis of the same parasite-prey interactions. The genomes of our mycoparasitic *Pythium* species were compared to those of nine other oomycete pathogens with different host and lifestyle ranges, to test the hypothesis that like their fungal counterparts, mycoparasitic oomycetes have expanded CAZyomes and that deployment of these cell wall degrading enzymes is also important for mycoparasitic oomycete-oomycete or oomycete-fungal interactions.

Our findings suggest that an expanded CAZyome may be one hallmark of mycoparasitism in eukaryotic microbes. Several of the CAZy encoding gene families appear to have been acquired by in mycoparasitic oomycetes through horizontal gene transfer. Our data also suggests that some CAZy-encoding genes act as pathogenicity factors, residing in similar genomic locations and with the potential to undergo rapid evolution in a similar manner to effector genes from phytopathogenic oomycetes.

## Results and Discussion

### The CAZyme of Mycoparasitic *Pythium* Species Contains Unique Features

Whilst it is well known that mycoparasitic fungi such as species within the *Trichoderma* Genus secrete an array of cell wall degrading enzymes during mycoparasitism of their prey, there is more limited information on the molecular mechanisms of mycoparasitism in the oomycete lineage. Previously several CAZyme-encoding genes were found to be expressed by *P. oligandrum* when interacting with tissue from the oomycete prey species, *Ph. infestans* (Horner et al., 2012). However, no comprehensive analysis of the CAZyome of *P. oligandrum* or *P. periplocum* has so far been carried out. In order to identify the key features and evolution of the mycoparasitic *Pythium* CAZyome, putative CAZyme encoding genes from *P. oligandrum*, *P. periplocum* and 11 other oomycete species were predicted using the dbCAN CAZy annotation pipeline (Yin et al., 2012). We first evaluated genome completeness in terms of the expected gene content of the thirteen species used for the comparison by carrying out a Benchmarking Universal Single-Copy Orthologs (BUSCO) analysis (Seppey et al., 2019). Complete and single copy BUSCO groups account for at least 80% of each genome (Supplementary Figure S1), and thus we concluded that the genome completeness of each organism was comparable. The CAZyme complement of the genomes of the mycoparasitic species *Pythium oligandrum* (Kushwaha et al., 2017a) and *Pythium periplocum* (Kushwaha et al., 2017b), was compared to those from the plant pathogens: *Pythium ultimum* (Lévesque et al., 2010), *Pythium aphanodermatum*, *Pythium arrhenomanes*, *Pythium iwayamai*, *Pythium irregulare*, *Phytopythium vexans* (Adhikari et al., 2013), *Phytophthora ramorum* (Tyler et al., 2006), *Phytophthora infestans* (Haas et al., 2009), and *Phytophthora capsica* (Lamour et al., 2012), and the human pathogen *Pythium insidiosum* (Rujirawat et al., 2015).

**FIGURE 1:**
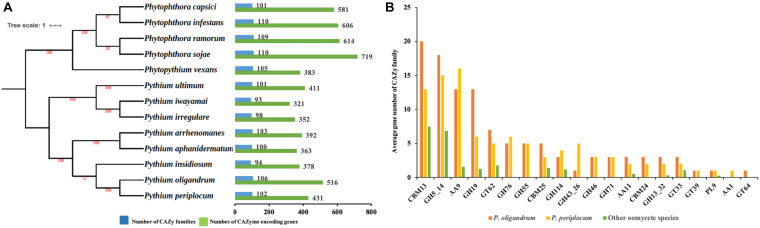
The CAZyme of two mycoparasitic *Pythium* species. **(A)** Species tree of oomycetes in this study and distribution of CAZy proteins. The species tree was constructed using the maximum likelihood method with 1,000 bootstraps based on a concatenated alignment of housekeeping genes identified by CEGMA analysis. Green bars indicate the total number of CAZyme encoding genes. Blue bars indicate the number of CAZy families present. **(B)** Average gene number of CAZy families unique or expanding in mycoparasitic *Pythium*. CAZy families were identified as unique or expanding in mycoparasitic *Pythium* species compare to other oomycetes by a *T*-test analysis.

We predicted 516 proteins in the CAZyome of *P. oligandrum*, 431 proteins in that of *P. periplocum*, and 321–719 proteins in the other oomycete species (Figure 1A). The total number of CAZy families predicted in *P. oligandrum* was 106, with 102 predicted in *P. periplocum*. The total number of CAZy families in the other oomycetes ranged from 93 to 110 (Figure 1A). These numbers are in line with previously reported predictions of the oomycete CAZyome (Ospina-Giraldo et al., 2010; Adhikari et al., 2013), indicating that our predictions are reliable. Within the *Pythium* genus, the total number of CAZy encoding genes was higher on average in the mycoparasitic *Pythium* species compared to the plant pathogenic *Pythium* species. However, since phytopathogenic *Phytophthora* species exhibit higher numbers of total CAZy genes, there is no significant difference within the oomycete lineage in the total numbers of CAZy encoding genes or in the total number of CAZy families per species.

To identify redundancy within the dataset and to compare CAZymes between the species, a comparison of the average gene member count per CAZy family was performed. Twenty CAZy families were identified as either expanded or unique within the mycoparasitic *Pythium* genomes when compared to the other species used in the analysis (*p* < 0.05, *T*-test; Figure 1B). These families include: three Auxiliary Activity families (AA1, AA11, AA9), three Carbohydrate-Binding Module families (CBM13, CBM24, CBM25), nine Glycoside Hydrolase families (GH114, GH13_32, GH19, GH43_26, GH46, GH5_14, GH55, GH71, GH76), four Glycosyl Transferase families (GT33, GT39, GT62, GT64), and one Polysaccharide Lyase family (PL9).

Alongside these analyses, we also mined RNA sequencing (RNA-Seq) data from the interactions between *P. oligandrum* with *Ph. infestans. P. periplocum* with *Ph. infestans* and from *P. periplocum* with *Botrytis cinerea*, to check the expression of these and other CAZy families. As well as the detailed analysis of the CAZy families described below, this also allowed us to identify differentially expressed genes during mycoparasitism of oomycete prey that were not encoded by families expanded in the mycoparasites, but that nonetheless may play a role in parasite-prey interactions (Supplementary Figure S2).

Since CAZy families with a putative role in the degradation of fungal or oomycete cell walls were predominantly expanded in the mycoparasitic oomycetes, we therefore focused the rest of our studies on carbohydrate binding and the metabolism of, cellulose, glucan, chitin and hemicelluloses, the major constituents of the cell walls of these prey species. Thus, eight CAZy families namely, AA9, GH5_14, GH55, GH71, GH19, GH46, GH76, and GH43_26 were chosen for detailed analysis. Among them, representatives of the CAZy families GH55, GH71, GH46, GH76, and GH43_26 were only detected in mycoparasitic *Pythium* species and were absent in the other oomycete genomes tested (Figure 1B and Supplementary Table S2) and thus appear to be unique components of the mycoparasitic oomycete CAZyme. Moreover, we also performed ortholog clustering using OrthoFinder (Emms and Kelly, 2019), which provides ortholog relationship analysis within each of the CAZy families mentioned above (Supplementary Table S3).

### Cellulose Metabolism

The major structural component of the oomycete cell wall is cellulose (Sietsma et al., 1969) and cellulose has been implicated in the maintenance of correct cell morphology and the production of appressoria (Grenville-Briggs et al., 2008). Penetration of plant tissue also requires active cellulose synthesis in the phytopathogenic oomycete, *Ph. infestans* (Grenville-Briggs et al., 2008). Homologs of the predicted cellulose synthase encoding genes [the CesA genes from the glycoside hydrolase family 2 (GT2)], previously identified in *Ph. infestans* (Grenville-Briggs et al., 2008) were identified in both *P. oligandrum* and *P. periplocum*, and this family was not expanded in the mycoparasites compared to the phytopathogens, although *P. oligandrum* contains one extra copy of the gene encoding CesA3 compared to *P. periplocum*, and the gene encoding CesA1 appears to be absent from *P. periplocum* (Supplementary Figure S7A). Although the CesA family is not expanded in the mycoparasites, genes encoding all eight CesA proteins identified in the mycoparasitic *Pythium* species, and a further GT2 protein from *P. periplocum*, were differentially expressed during interactions of the mycoparasite with *Ph. infestans* (Supplementary Figures S2A, S7B). Thus, although, it is not known whether appressorium production is also a significant component of oomycete mycoparasitism, the synthesis of cellulose may of general importance for growth during mycoparasitic interactions.

We hypothesize that cellulose degradation enzymes may be important pathogenicity determinants in the mycoparasitic *Pythium* species, since they are part of the cell wall of oomycete prey. Two families of genes encoding enzymes predicted to be involved in the degradation of cellulose were expanded in the mycoparasitic species, namely AA9 and GH5_14.

**FIGURE 2:**
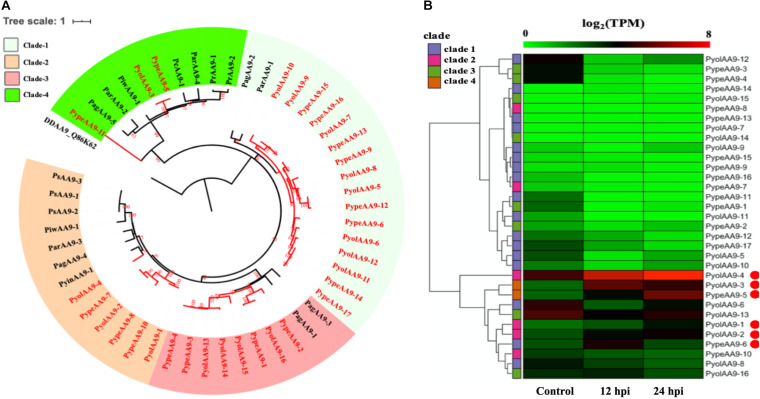
Analysis of auxiliary activity family 9 (AA9). **(A)** Phylogenetic tree of AA9 proteins identified in oomycete species. Maximum likelihood tree, with 1,000 bootstraps (values displayed per branch). DDAA9_Q86K62 (an AA10 protein verified in *Dictyostelium discoideu*m) was used as an outgroup. AA9 proteins identified in *P. oligandrum* (Pyol) and *P. periplocum* (Pype)are marked in red. **(B)** RNAseq Expression profiles of AA9 genes from *P. oligandrum* and *P. periplocum* during interactions with *Ph. infestans* during *in vitro* growth (control) or at 12 or 24 h post interaction (hpi). The phylogeny of each protein is marked using colored bars to represent each clade. The genes with the highest expression level during the interactions with *Ph. infestans* are marked with red circles. Expression levels are expressed as the log_2_ fold change of transcripts per million (TPM), per gene.

The auxiliary activity family 9 (AA9), proteins are lytic polysaccharide monooxygenases that are predominantly found in fungi. The AA9 family was significantly expanded in the mycoparasitic *Pythium* genomes compared to the other oomycete species studied. 16 AA9 proteins were predicted in *P. oligandrum* and 17 in *P. periplocum*. A range of one to five AA9 proteins were identified in plant pathogenic *Pythium* species, one AA9 protein was identified in the human infecting *Pythium*, and six AA9 proteins in *Phytophthora* species. Q86K62, an Auxiliary Activities 10 (AA10) protein in *Dictyostelium discoideum*, was used as outgroup to produce an AA9 phylogenetic tree, which consists of four clades (Figure 2B). Among clades, 17 mycoparasitic *Pythium* AA9 proteins are present in clade 1 (eight *P. oligandrum* and nine *P. periplocum*). Eight AA9 proteins are present in clade 3 (four from *P. oligandrum* and four from *P. periplocum*). Therefore, expansion of AA9 family in mycoparasitic *Pythium* appears to occur primarily in clade 1 and clade 3.

Previous studies have identified a “two-speed” architecture in the genomes of plant pathogenic oomycetes, in which the gene sparse regions exhibit higher plasticity, and hence drive adaptive evolution, that the gene dense regions (Raffaele and Kamoun, 2012). Pathogenicity determinants such as the RxLR effectors from phytopathogenic oomycetes tend to occupy regions that are gene poor, but repeat rich, facilitating rapid evolution of those regions (Dong et al., 2015). Detailed analysis of the *Ph. infestans* genome (Haas et al., 2009) postulated that accelerated effector evolution is driven by tandem gene duplication and homologous recombination. Based on gene density analysis, the majority of the *P. oligandrum* AA9 genes, encoding AA9 proteins assigned to clade 1, are located within the gene sparse regions (Supplementary Figure S3A). AA9 genes encoding clade 3 AA9 proteins, however, are present in the gene dense regions. Genome location analysis of the AA9 genes within the *P. oligandrum* genome shows that they cluster in two tandem arrays, consisting of AA9 genes encoding AA9 proteins of clade 1 and clade 3, respectively (Supplementary Figure S4A), suggesting that members of the AA9 family may be under rapid evolution in mycoparasitic oomycetes and further, may be important pathogenicity determinants for this group of organisms.

Using normalized TPM read counts from RNA-Seq data of the interaction between either *P. oligandrum* or *P. periplocum* and the prey *Ph. infestans*, we found seven AA9 genes were highly expressed specifically in the interactions between host and prey (Figure 2B). Among them, *PyolAA9-2*, *PyolAA9-3*, and *PyolAA9-4* were significantly up-regulated at 12 and 24 hpi of the interaction and may be of general importance during parasite-prey interactions. *PypeAA9-6* was only significantly up-regulated at 12 hpi, so may act predominantly in the early stages of mycoparasitism and thus could be described as a putative pathogenicity factor. *PypeAA9-5* and *PyolAA9-1*were significantly up-regulated at 24 hpi and therefore play an important role in the later stage of mycoparasitism. *PyolAA9-13* was comparatively highly expressed during both *in vitro* growth of both *Pythium* species as well as during interactions with oomycete prey and thus may be important for remodeling of the parasite cell wall during both vegetative growth and feeding.

We were able to design specific primers for qRT-PCR amplification of three of the AA9 genes, namely *PyolAA9-3*, *PyolAA9-13*, and *PyolAA9-16* (Figure 10A). Both genes showed an induction during mycoparasitism of *Ph. infestans*, with *PyolAA9-3* displaying the highest relative expression at 6 h, during the early colonization of *Ph. infestans* tissue. *PyolAA9-13* was induced at 6 h and continued to show an elevated expression relative to *in vitro* growth levels from 6 to 48 h, with a peak at 36 h. *PyolAA9-16* displayed constitutive levels of expression during both vegetative growth and the first 6 hpi which then reduced during the later stages of the interaction. These results suggest that AA9 family proteins have a role in mycoparasitism of *Ph. infestans* by mycoparasitic *Pythium* species.

**FIGURE 3:**
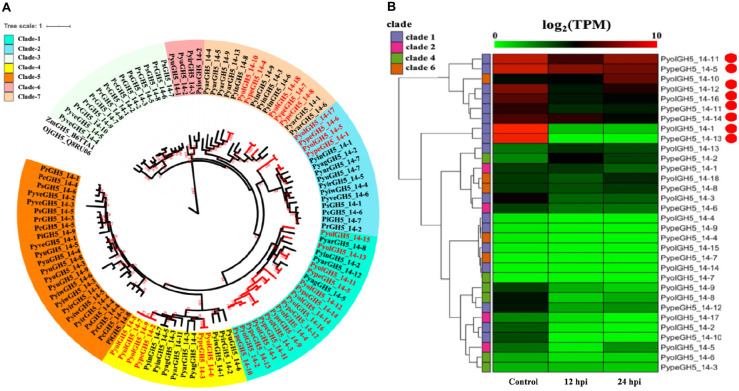
Analysis of glycoside hydrolase family 5_14 (GH5_14). **(A)** Phylogenetic tree of GH5_14 proteins identified in oomycete species. Maximum likelihood tree, with 1,000 bootstraps (values displayed per branch). ZmGH5_B6TTA1 and OjGH5_Q8RU06 (GH5_14 proteins verified in plant species) were used as outgroups. GH5_14 proteins identified in *P. oligandrum* (Pyol) and *P. periplocum* (Pype) are marked in red. **(B)** RNAseq Expression profiles of GH5_14 genes from *P. oligandrum* and *P. periplocum* during *in vitro* growth or interactions with *Ph. infestans* and 12 or 24 h post interaction. The phylogeny of each protein is marked using colored bars to represent each clade. The genes with the highest expression level during the interactions with *Ph. infestans* are marked with red circles. Expression levels are expressed as the log_2_ fold change of transcripts per million (TPM), per gene.

An AA9 gene from the saprotrophic fungus *Chaetomium globosum* has previously been shown to promote cellulose hydrolysis by GH5_14 family cellulases and also exhibits the same synergistic effect in xylan hydrolysis (Kim et al., 2015, 2016). Since we also found the GH5_14 (predicted enzyme activity as endo-1,4-β-D-glucanases/cellulases (EC3.2.1.4), to be significantly expanded in the mycoparasitic *Pythium* genomes, compared to their phytopathogenic counterparts, we also checked for the presence of the genes encoding the GH5_14 family in gene sparse regions of the genome and for duplication events. The GH5_14 family comprises 18 proteins in *P. oligandrum* and 15 in *P. periplocum*. The phylogenic tree of GH5_14 proteins from all oomycetes in this study can be divided into seven clades (Figure 3A). Two plant GH5_14 proteins, ZmGH5_B6TTA1 and OjGH5_Q8RU06, were used as outgroups. GH5_14 proteins from the mycoparasitic *Pythium* species are present in clade 1, clade 2, clade 4, and clade 6. Notably, most of the GH5_14 proteins from the mycoparasitic *Pythiums* are present in clade 1 (10 from *P. oligandrum* and seven from *P. periplocum*). Thus, the expansion of the GH5_14 family occurred primarily in clade 1. Furthermore, genes encoding those proteins in clade 1 reside in the gene sparse regions of the genome and are located in tandem arrays, suggesting that these two families of associated genes are undergoing rapid evolution (Supplementary Figures S3B, S4B) and thus may also be important as pathogenicity determinants.

Using normalized TPM read counts from RNA-Seq data of the interaction between either *P. oligandrum* or *P. periplocum* and the prey, *Ph. infestans*, we could determine that eight putative cellulase, GH5_14 genes encoding proteins assigned to clade 1, were highly expressed during mycoparasite-prey interactions, or during *in vitro* growth (Figure 3B), among them, two genes, *PyolGH5_14-11* and *PypeGH5_14-5*, showed near constitutive expression levels in all conditions tested, suggesting a role in the remodeling of the mycoparasitc cell wall during all phases of growth. Four genes, *PypeGH5_14-11*, *PyolGH5_14-12*, *PypeGH5_14-14*, *PypeGH5_14-16*, were highly expressed during *in vitro* growth and slightly down-regulated during interactions with the prey species. *PyolGH5_14-1* and *PypeGH5_14-13*, were highly expressed mainly during *in vitro* growth. This suggests the latter two genes have a role in the modification of the cell wall of the mycoparasite during vegetative growth, and little to no role in mycoparasitism. The genes encoding GH5_14 proteins in clade 2, clade 4, and clade 6 were all expressed at relatively low levels during *in vitro* growth, and/or during the interaction with *Ph. infestans*. However, transcripts from *PyolGH5_14-10*, assigned to clade 6, were highly abundant in the *Ph. infestans* interaction at 24 hpi.

Detailed expression analysis of a time course of *Ph. infestans* infection by *P. oligandrum* revealed that, of the genes tested, *PyolGH5_14-16* was highly expressed during mycoparasitism, peaking at 36 h with a relative expression level greater than 300 times that of the *in vitro* expression (Figure 10A). Furthermore, data from three independent biological replicates shows that the expression of this gene oscillates between higher and lower expression over the interaction time course. A temporal switching of gene expression may be a response to the availability of new host hyphae, reflecting enzyme production only as and when needed, e.g., through digestion of a single hypha at a time. Alternatively, this could be part of a mechanism to avoid accidental self-damage. The fungus *Trichoderma reesei*, is a prolific producer of cellulases, and as such is a model organism for industrial production of several CAZy enzymes involved in cell wall degradation. Major cellulase transcription factor genes have been identified that show differential regulation under light or dark conditions and photoreceptors play an important role in regulation of nutritional uptake in this organism (Schmoll, 2018). *PyolGH5_14-16* transcript abundance peaks occurred during the night sampling (12 and 36 h) of the interaction time course and this pattern is reminiscent of a circadian rhythm. However, this may also reflect gene induction under the somewhat artificial *in vitro* conditions under which the experiments were performed and thus investigation of the expression of CWDEs under natural or field conditions would be interesting for the future. *PyolGH5_14-1* and *PyolGH5_14-12* show a decreased expression during mycoparasitism, relative to *in vitro* levels, indicating a potential role predominantly during vegetative growth. Neither the GH6 family of putative cellobiohydrolases (EC 3.2.1.91) or the GH5_20 family of endo-β-1,4-glucanases/cellulases (EC 3.2.1.4) were expanded in the mycoparasitic oomycetes, however, five GH6 family genes from *P. oligandrum* were differentially expressed (significantly upregulated) during the early stages of interactions with *Ph. infestans*. whilst five GH5_20 genes were down-regulated (Supplementary Figure S2A). Four GH5_20 genes from *P. periplocum* were also down-regulated at the same time point, as were three GH3 β-glucosidases (EC 3.2.1.21) (Supplementary Figure S2B), indicating these may be used to cleave celluloses from other substrates, or involved in other phases of the mycoparasitic lifecycle. Genes encoding 10 putative GH17 family endo-1,3-β-glucosidases (EC 3.2.1.39) from *P. oligandrum* were upregulated during the early stages of interactions with *Ph. infestans* five from *P. periplocum* were upregulated in the later stages of mycoparasitism (Supplementary Figure S2), indicating that the different mycoparasites may use a progression of different cellulose degrading enzymes at different time points in the mycoparasitic life cycle.

Thus, the metabolism of cellulose is likely to be important for both vegetative oomycete growth and for mycoparasitism of oomycete prey and several of the genes involved in this process display the genomic hallmarks of pathogenicity factors undergoing rapid evolution.

### Glucan Metabolism

**FIGURE 4:**
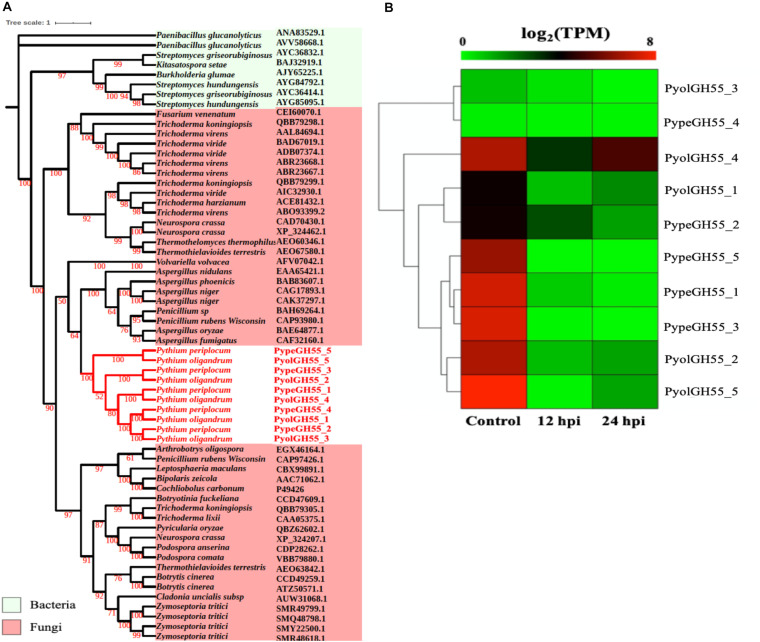
Analysis of glycoside hydrolase family 55 (GH55). **(A)** Phylogenic tree of GH55 proteins identified in *P. oligandrum* and *P. periplocum*. Maximum likelihood tree, with 1,000 bootstraps (values displayed per branch). GH55 proteins identified in *P. oligandrum* (Pyol) and *P. periplocum* (Pype) are shown in red. **(B)** RNAseq Expression profiles of GH55 genes detected in *P. oligandrum* and *P. periplocum* during *in vitro* growth or interactions with *Ph. infestans* at 12 or 24 h post interaction. Expression levels are expressed as the log_2_ fold change of transcripts per million (TPM), per gene.

Glucans are structurally related to cellulose and are another important component of the oomycete cell wall. In contrast to cellulose, glucans are largely absent from plant cell walls and thus dissecting the occurrence and evolution of enzymes involved in the degradation of glucans may provide unique insights into the evolution of the mycoparasitic oomycete species. GH55 CAZy family members are predicted to function as exo-β-1,3-glucanases (EC 3.2.1.58 degrading glucans containing β-1,3-linkages as well as participating in the hydrolysis of laminarin, a component of fungal cell walls (Bara et al., 2003). Thus, genes in this class may potentially target both oomycete and fungal prey. Genes assigned to the CAZy family GH55 were found only in the mycoparasitic *Pythium* and were absent in the other oomycete genomes tested, indicating that they might be important for mycoparasitism. We found genes encoding five GH55 proteins each in *P. oligandrum* and *P. periplocum*. Phylogenetic analysis shows that oomycete GH55 proteins are most closely related to those from a variety of fungal species (Figure 4A). It has recently been shown that almost half of the cell wall degrading carbohydrate active enzymes found in mycoparasitic *Trichoderma* species were obtained via horizontal gene transfer, a process by which genetic material may be transferred between distinct evolutionary lineages, either cross-Kingdom or within Kingdom (Doolittle, 1999; Andersson, 2009), from plant associated filamentous fungi (Druzhinina et al., 2018). Our data supports the hypothesis that *P. oligandrum* and *P. periplocum* have developed the ability to be mycoparasitic, in part, through the acquisition of CWDEs from the GH55 family, via horizontal gene transfer from filamentous fungi. The flanking regions of mycoparasitic *Pythium* GH55 genes show conserved collinearity with regions of the *P. ultimum* genome (Supplementary Figure S5A), although GH55 genes are absent in *P. ultimum*. Therefore, mycoparasitic *Pythium* GH55 genes appear to reside within conserved regions of their genomes and may potentially have been inserted there via horizontal gene transfer from fungi.

Analysis of normalized TPM read counts from the RNA-Seq interaction libraries, revealed two different expression patterns. Six GH55 genes, (*PyolGH55_4*, *PypeGH55_5*, *PypeGH55_1*, *PypeGH55_3*, *PyolGH55_2*, *PyolGH55_5*), were highly expressed during *in vitro* growth of and significantly down-regulated in the interaction stages (Figure 4B). Notably, *PyolGH55_4* was highly expressed both during *in vitro* growth and at the later stage of the interaction between *P. oligandrum Ph. infestans*. Four GH55 genes (*PyolGH55_3*, *PypeGH55_4*, *PyolGH55_1*, *PypeGH55_2*), show a low level of expression in all stages tested, suggesting that they do not have a major role during *in vitro* growth or in the interaction with *Ph. infestans* as a prey species. They may, however, be expressed more highly in other growth stages or in the interaction with other prey species.

**FIGURE 5:**
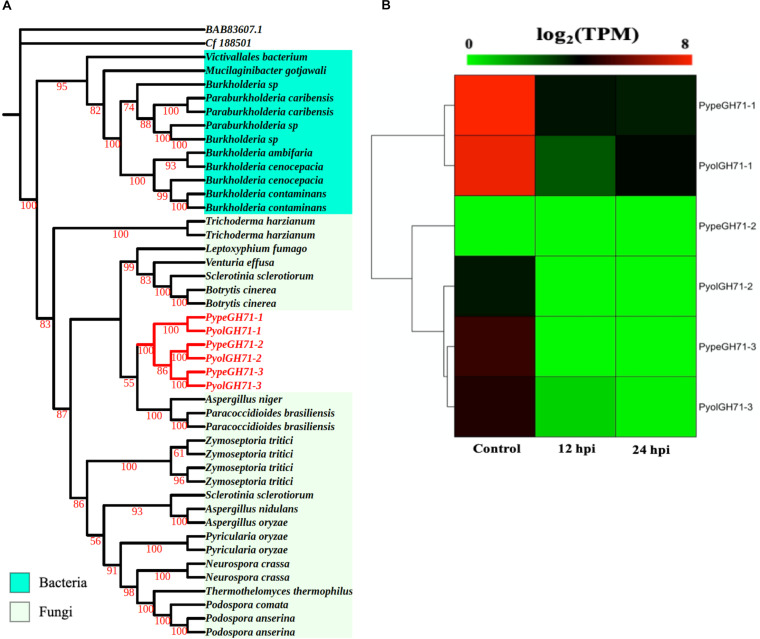
Analysis of glycoside hydrolase family 71 (GH71). **(A)** Phylogenic tree of GH71proteins identified in *P. oligandrum* and *P. periplocum*. Maximum likelihood tree, with 1,000 bootstraps (values displayed per branch). GH71 proteins identified in *P. oligandrum* (Pyol) and *P. periplocum* (Pype) are shown in red. **(B)** RNAseq Expression profiles of GH71 genes detected in *P. oligandrum* and *P. periplocum* during *in vitro* growth or interactions with *Ph. infestans* at 12 or 24 h post interaction. Expression levels are expressed as the log_2_ fold change of transcripts per million (TPM), per gene.

Like the GH55 family, members of GH71 family were only identified in mycoparasitic *Pythium* among oomycete species in this study. GH71 proteins have a predicted enzymatic activity as α-1,3-glucanases (EC 3.2.1.59). We found three GH71 proteins in each of the mycoparasitic species *P. oligandrum* and *P. periplocum*, respectively. Phylogenetic analysis groups these genes within a branch of fungal GH71 proteins (Figure 5A). Thus, this suggests that the GH71 genes from the mycoparasitic *Pythium* species may also have been obtained via horizontal transfer from fungi. Two of the genes, *PyolGH71-1* and *PypeGH71-1*, are highly expressed during *in vitro* growth and are significantly down-regulated during the interaction with *Ph. infestans* (Figure 5B). The remaining members of this family show low levels of expression in the stages tested in this study, which is a similar expression profile to the GH55 glucanase family. *PyolGH71-*1 showed high relative expression levels at 36 and 48 h of the interaction with *Ph. infestans*, suggesting either a role in degradation of prey glucans that are exposed later during mycoparasitism or a role in *P. oligandrum* cell wall rearrangement and/or growth, after nutrient uptake from the prey.

We next used the GH55 and GH71 protein sequences detected in *P. oligandrum* and *P. periplocum* in blastp searches against the NR database, excluding sequences from *P. oligandrum* and *P. periplocum* themselves. Compiling the resultant data as a phylogenetic tree, further confirms that GH55 and GH71 proteins appear to have been transferred horizontally from Ascomycete fungi to the oomycetes (Supplementary Figure S8). Moreover, we also made Hidden Markov Model (HMM) searches against the genomes of other available oomycetes, namely, *Hyaloperonospora arabidopsidis*, *Albugo laibachii*, *Saprolegnia parasitica*, *Saprolegnia diclina*, *Phytophthora parasitica*, and *Pythium brassicum*. However, we could not find any GH55 and GH71-like genes in these species, and thus conclude that they are absent in other oomycetes, for which we have genome data. Therefore, it appears that horizontal gene transfer of GH55 and GH71 genes occurred within the latest common ancestor of *P. oligandrum*.

### Chitin Metabolism

**FIGURE 6:**
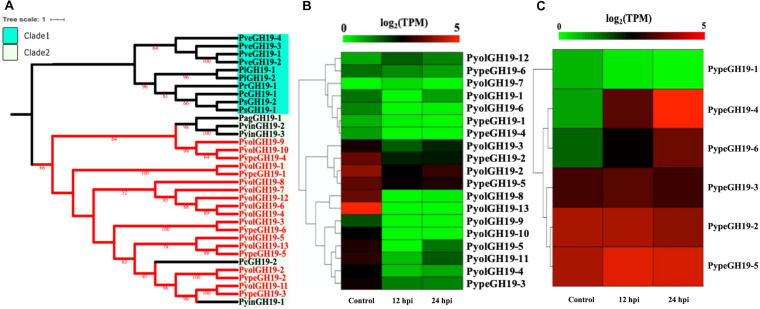
Analysis of glycoside hydrolase family 19 (GH19). **(A)** Phylogenetic tree of GH19 proteins identified in oomycete species. Maximum-likelihood method with 1,000 bootstraps (values displayed per branch) was used to construct tree based on alignment of GH19 proteins. GH19 proteins identified in *P. oligandrum* (Pyol) and *P. periplocum* (Pype) are shown in red. **(B)** RNAseq Expression profile of GH19 genes detected in *P. oligandrum* and *P. periplocum* during *in vitro* growth or interactions with *Ph. infestans* at 12 or 24 h post interaction. Expression levels are expressed as the log_2_ fold change of transcripts per million (TPM), per gene. **(C)** RNAseq Expression profile of GH19 genes detected in *P. periplocum* during *in vitro* growth or interactions with *B. cinerea* at 12 or 24 h post interaction. Expression levels are expressed as the log_2_ fold change of transcripts per million (TPM), per gene.

Members of the GH19 family, with predicted activity as chitinases (EC 3.2.1.14), are abundant in mycoparasitic *Pythium* species (Figure 6A). Here we found 13 GH19 proteins *in P. oligandrum* and six in *P. periplocum*. A range of one to four GH19 proteins were found in other oomycetes in this study. As shown in Figure 6A, GH19 proteins can be divided into only two clades. GH19 proteins from the mycoparasitic *Pythium* species were only present in clade 2, which also includes one GH19 proteins from each of the plant pathogens *P. aphanidermatum*, and *Ph. capsici* and three genes from the human and animal pathogen *P. insidiosum*. Based on gene density analysis, most of the GH19 genes identified, are predicted to be present in the gene sparse regions of the genomes of both *P. oligandrum* and *P. periplocum* (Supplementary Figures S3C,D). Moreover, one tandem array, consisting of genes *PyolGH19-3* to *PyolGH19-10*, is present in the genome of *P. oligandrum* (Supplementary Figure S4C), suggesting that these genes are able to undergo rapid evolutionary change, and may be important pathogenicity factors in fungal mycoparasitism.

Analysis of normalized TPM read counts from our RNA-Seq interaction libraries reveals that GH19 genes (*PypeGH19-2*, *PyolGH19-2*, *PypeGH19-5*, *PyolGH19-8*, *PyolGH19-13*), were highly expressed during *in vitro* growth and significantly down-regulated during the interaction with the prey oomycete *Ph. infestans* (Figure 6B). Thus, GH19 genes seem to be dispensable for interactions between mycoparasitic *Pythium* species and *Ph. infestans*, which has a cellulosic cell wall and no detectable chitin (Sietsma et al., 1969; Mélida et al., 2013). To test the hypothesis that GH19 proteins are used for parasitism of fungal prey, we mined our sequenced transcriptomes of interactions between *P. periplocum* and the gray mold fungus, *Botrytis cinerea*. Three *P. periplocum* GH19 genes (*PypeGH19-2*, *PypeGH19-3*, and *PypeGH19-5*), were highly expressed during both *in vitro* growth and during the interactions between *P. periplocum* and *B. cinerea.* Two *P. periplocum* GH19 genes (*PypeGH19-4*, and *PypeGH19-6*), were highly expressed solely during interactions between *P. periplocum* and *B. cinerea* and *PypeGH19-1* induction was not detected in any of the conditions we tested (Figure 6C).

To confirm the role of GH19 family members in mycoparasitism of *B. cinerea*, we performed qRT-PCR analysis of selected GH19 genes during the early stages of parasitism by either *P. periplocum* or *P. oligandrum*. Four *P. periplocum* GH19 genes, (*PypeGH19-1*, *PypeGH19-4*, *PypeGH19-5*, *PypeGH19-6*), were significantly upregulated (between two and fifteen -fold change) at 12 and 24 h of interactions with *B. cinerea.* Six *P. oligandrum* GH19 genes (*PyolGH19-1*, *PyolGH19-2*, *PyolGH19-3*, *PyolGH19-8*, *PyolGH19-9*, *PyolGH19-11*), were significantly induced (7–1,300-fold change) during the interaction with *B. cinerea*. Thus, chitinase gene expression is likely to be important for mycoparasitism of fungal prey and the mycoparasitic *Pythium* species are able to sense the cell wall components of their prey and adjust the expression of their CWDEs accordingly, since these genes are not expressed in the presence of a non-chitinous prey species.

**FIGURE 7:**
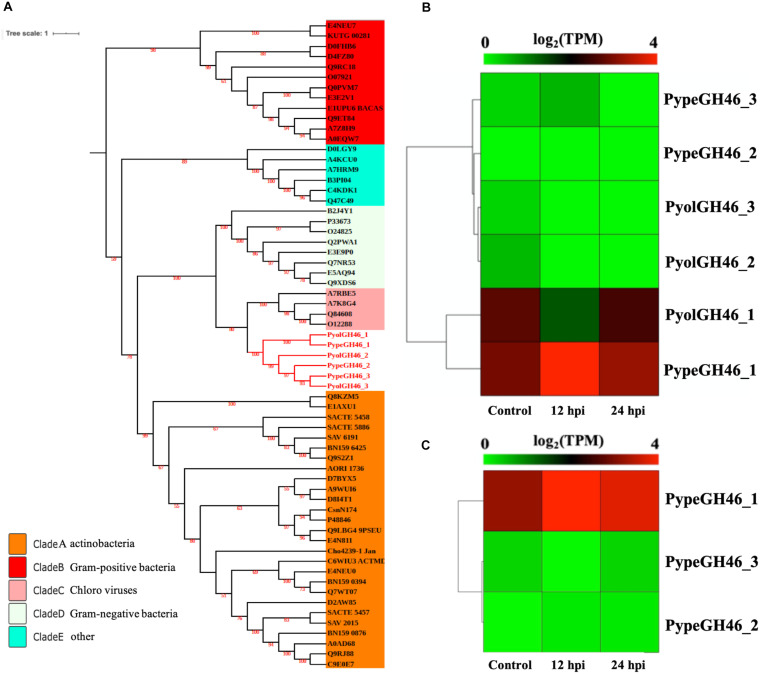
Analysis of glycoside hydrolase family 46 (GH46). **(A)** Phylogenetic tree of GH46 proteins identified in oomycete species. Maximum likelihood tree, with 1,000 bootstraps (values displayed per branch). GH46 proteins identified in *P. oligandrum* (Pyol) and *P. periplocum* (Pype) are marked in red. **(B)** RNAseq expression profile of GH46 genes detected in *P. oligandrum* and *P. periplocum* during *in vitro* growth or during interactions with *Ph. infestans* at 12 or 24 h post interaction. Expression levels are expressed as the log_2_ fold change of transcripts per million (TPM), per gene. **(C)** RNAseq Expression profile of GH46 genes detected in *P. periplocum* during *in vitro* growth or interacting with *B. cinerea* at 12 or 24 h post interaction. Expression levels are expressed as the log_2_ fold change of transcripts per million (TPM), per gene.

The GH46 family (EC.3.2.1.132) is predicted to have chitosanase activity and was detected only in the mycoparasitic *Pythium* and not in the other oomycete species screened in this study. Chitosan is a cationic polysaccharide composed of β-1,4-linked D-glucosamine (GlcN) that is derived from chitin, with chitosanase being the major substrate-specific enzyme that acts on the β-1,4-glycosidic linkages of chitosan (Sun et al., 2018). We detected three GH46 genes in both *P. oligandrum* and *P. periplocum* respectively. To explore the phylogenic relationship of the mycoparasitic *Pythium* GH46 genes, we retrieved GH46 proteins from a diverse range of species (Viens et al., 2015). As Figure 7A shows, the phylogenic tree of the GH46 family is divided into five clades, as previously reported (Viens et al., 2015). Mycoparasitic *Pythium* GH46 proteins are present in clade C, which includes GH46 proteins from *Chlorella virus*. Thus, we inferred that mycoparasitic *Pythium* may have been obtained GH46 genes by horizontal transferred from a viral donor. Genes neighboring the GH46 gene family in the mycoparasitic *Pythium* show collinearity with regions of the *P. ultimum* genome (Supplementary Figure S5B). Therefore, it would appear that the mycoparasitic *Pythium* GH46 genes were transferred into conserved regions of their genomes.

Interestingly, transcripts of one GH46 gene (*PypeGH46-1*) were highly abundant, relative to *in vitro* levels, during the parasitism of both *Ph. infestans* and *B. cinerea* by *P. periplocum.* One gene from *P. oligandrumm (PyolGH46-1)* was also expressed during parasitism of *Ph. infestans* (Figures 7B,C). Quantitative RT-PCR assays of GH46 genes during the early stages of interactions of either *P. periplocum* or *P. oligandrum* with *B.* cinerea reveal five genes (*PyolGH46-1*, *PyolGH46-2*, *PyolGH46-3*, *PypeGH46-1*, and *PypeGH46-3*), that are induced between 2 and 50-fold during parasitism of this fungus (Figure 10).

It has long been thought that the oomycetes are predominantly cellulosic in contrast to the chitinous fungi with whom they share many ecological niches. However detailed analysis of the structural carbohydrates in the cell wall of several plant and fish pathogenic oomycetes has identified three types of cell wall-based differences in structural carbohydrates within different oomycete Genera (Mélida et al., 2013). Type I contains glucuronic acid and mannose and no N-acetylglucosamine (GlcNAc), type II is characterized by cross linking between cellulose and 1,3,β glucans and up to 5% GlcNAc and the third type contains the highest GlcNAc content along with unusual carbohydrates (Mélida et al., 2013). This analysis did not include any of the mycoparasitic oomycetes or members of the *Pythium* genus though, so it is not clear which cell wall type *P. oligandrum* or *P. periplocum* contain, although based on our data showing that chitin metabolism-linked genes are expressed under *in vitro* conditions, we hypothesize that the mycoparasitic oomycetes may be modulating related carbohydrates or chito-oligosaccharides within their own cell walls. Alternatively, these genes may be expressed *in vitro* in preparation for degradation of prey cell walls, or in response to a depletion of nutrients.

### Hemicellulose Metabolism

**FIGURE 8:**
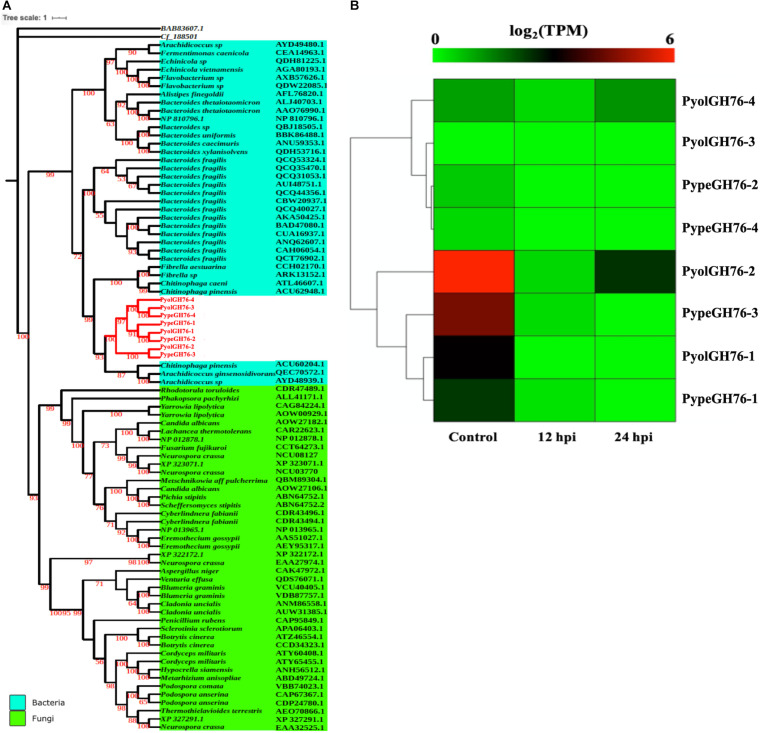
Analysis of glycoside hydrolase family 76 (GH76). **(A)** Phylogenetic tree of GH76 proteins identified in *P. oligandrum* and *P. periplocum* with GH76 proteins of bacteria and fungi. Maximum likelihood tree, with 1,000 bootstraps (values displayed per branch). GH76 proteins identified in *P. oligandrum* (Pyol) and *P. periplocum* (Pype) are marked in red. BAB83607.1 and Cf_188501 (GH55 proteins verified in fungal species) were used as an outgroup. **(B)** RNAseq Expression profile of GH76 genes detected in *P. oligandrum* and *P. periplocum* during *in vitro* growth or interactions with *Ph. infestans* at 12 or 24 h post interaction. Expression levels are expressed as the log_2_ fold change of transcripts per million (TPM), per gene.

Four GH76 family members were found each in both *P. oligandrum* and *P. periplocum* although GH76 genes were absent in the other oomycete species screened. GH76 proteins are annotated as α-1,6-mannanases (EC 3.2.1.101), targeting α-1,6-mannosidic linkages and thus participating in the deconstruction of fungal cell walls (Cuskin et al., 2015). The phylogenic analysis, with an exo-β-1,3-glucanase of GH55 proteins (BAB83607.1) as the outgroup, shows that mycoparasitic *Pythium* GH76 proteins group most closely in the GH76 proteins from bacterial species (Figure 8A). Thus, these genes may have been horizontally transferred to a common mycoparasitic *Pythium* ancestor from bacteria.

Analysis of normalized TPM read counts from our RNA-Seq libraries revealed that one of the *P. oligandrum* genes (*PyolGH76-2*) and one of the *P. periplocum* genes (*PypeGH76-3*) were highly expressed during *in vitro* growth (Figure 8B). However, there was little to no detectable differential expression during interactions with the prey species tested, meaning that these genes are dispensable for antagonism of the fungal and oomycete prey used in this study.

**FIGURE 9:**
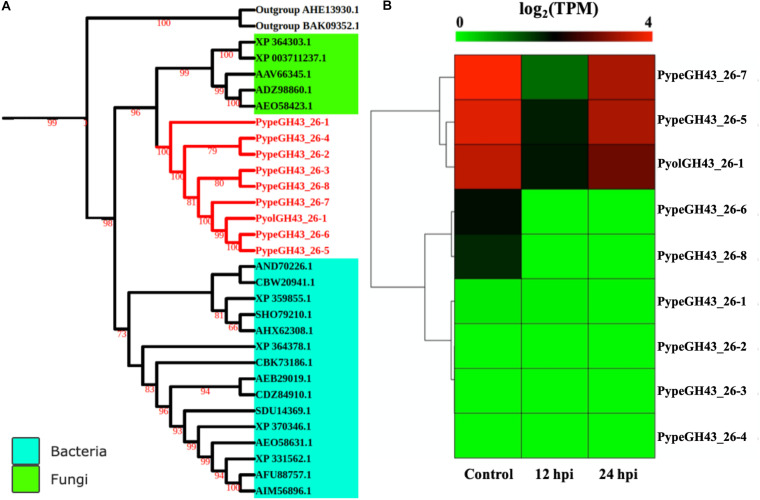
Analysis of glycoside hydrolase family 43_26 (GH43_26). **(A)** Phylogenetic tree of GH43_26 proteins identified in *P. oligandrum* and *P. periplocum* with GH43_26 proteins of bacteria and fungi retrieved from CAZy database. Maximum likelihood tree, with 1,000 bootstraps (values displayed per branch). GH43_26 proteins identified in *P. oligandrum* (Pyol) and *P. periplocum* (Pype) are marked in red. AHE13930.1 and BAK095352.1 (GH11 proteins verified in fungi species) were used as an outgroup. **(B)** RNAseq Expression profile of GH43_26 genes detected in *P. oligandrum* and *P. periplocum* during *in vitro* growth or at 12 or 24 h post interaction with *Ph. infestans*. Expression levels are expressed as the log_2_ fold change of transcripts per million (TPM), per gene.

GH43_26 genes were only identified in mycoparasitic *Pythium* among oomycetes in our study. Here we found one GH43_26 protein in *P. oligandrum* and eight GH43_26 proteins in *P. periplocum*. These genes have predicted activity as α-L-arabinofuranosidases (EC 3.2.1.55), which hydrolyze terminal non-reducing alpha-L-arabinofuranoside residues in alpha-L-arabinosides. Figure 9A shows that GH43_26 proteins from mycoparasitic *Pythiums* (red branch) group within a fungal GH43_26 clade (green branch). An endo-β-1,4-xylanase from the GH11 family (AHE13930.1) was used as an outgroup. This suggests that this gene family may have been horizontally transferred from fungi to mycoparasitic *Pythium*. Three of the GH43_26 genes (*PypeGH43_26-7*, *PypeGH43_26-5*, *PyolGH43_26-1*) were highly expressed both during *in vitro* growth and 24 hpi of the interaction with *Ph. infestans* indicating that they may be of some importance for growth of the mycoparasitic species themselves and of a lesser importance during mycoparasitism of oomycete prey (Figure 9B).

The GT71 family encodes genes with activity as α-mannosyltransferases (EC 2.4.1.-), and whilst this family was not expanded, nine GT71 genes were significantly upregulated in *P. oligandrum* during interactions with *Ph. infestans.* GH16 family members with predicted xyloglucanase activity (EC 3.2.1.151) were not expanded in the mycoparasitic oomycetes, however, three putative GH16 genes from *P. oligandrum* were upregulated during interactions with the prey oomycete *Ph. infestans* (Supplementary Figure S2). The family with the highest number of differentially expressed genes when interacting with the oomycete prey in both *P. oligandrum* and *P. periplocum* was CE1. Thirteen genes from *P. oligandrum* and fifteen genes from *P. periplocum* were upregulated during the early interactions with *Ph. infestans* (Supplementary Figure S2) and thus these family members may play a role in the degradation of hemicelluloses of oomycete origin. This Carbohydrate esterase family has diverse esterase functions including as acetyl xylan esterases (EC 3.1.1.72). GH30_1 family endo-β-1,4-xylanases from both mycoparasites were downregulated in the same interactions, indicating that they are not important in the interaction with oomycetes.

### Carbohydrate Binding Genes

Since destruction of cellulose appears to be important to oomycete-oomycete mycoparasitic interactions, and these *Pythium* species appear able to sense the major carbohydrate component of the prey cell wall and adapt their CWDE gene expression, we next investigated the presence and expression of cellulose binding module containing proteins, with the hypothesis that these may play a role in the sensing or binding of oomycete prey cells.

The cellulose-binding elicitor lectin (CBEL) family was found to contain two cellulose-binding domains (CBDs), belonging to the family 1 carbohydrate binding modules (CBM1). The CBM1 family is almost exclusively found only in fungi and oomycetes. In the phytopathogenic oomycetes, CBEL family members have been shown to have necrosis-inducing activity in host plants, and to bind host cellulose (Mateos et al., 1997; Gaulin et al., 2006). In a previous study of a small cDNA library of *P. oligandrum* interacting with dead tissue from *Ph. infestans*, a CBEL gene from *P. oligandrum* was shown to be upregulated during the interaction (Horner et al., 2012).

In the current study we found six CBEL proteins each in *P. oligandrum* and *P. periplocum*. A range of 2–10 CBEL proteins were detected in the other oomycete genomes screened. Supplementary Figure S6A shows that CBEL proteins were divided into four phylogenetic clades with a *Trichoderma reesei* exoglucanase used as an outgroup. Mycoparasitic *Pythium* CBEL proteins were only present in clade 4 (Supplementary Figure S6A). Based on domain architecture analysis, CBD (CBM1) domains are present in all putative CBEL proteins and PAN domains are only present in CBEL proteins grouped within clade 4. PAN domains are found in a diverse array of proteins and have been implicated in protein-protein or protein-carbohydrate interactions. Some of the mycoparasitic *Pythium* CBEL proteins are also predicted to contain other domains, including transglutaminase elicitors (TGase_elicitor), Leucine Rich Repeats (LRR_4 or LRR_8) and elicitin domains.

Analysis of normalized TPM read counts from our RNA-Seq libraries revealed that putative CBEL genes from *P. oligandrum* (*PyolCBEL_2*, *PyolCBEL_4*, *PyolCBEL_5*), and *P. periplocum* (*PypeCBEL_3*, *PypeCBEL_6*) were highly expressed during *in vitro* growth. These CBEL proteins are predicted to contain only a PAN domain and carbohydrate binding domain along with a signal peptide. Of these, *PyolCBEL_5* and *PypeCBEL_3* were also somewhat expressed during interactions with *Ph. infestans* (Supplementary Figure S6B). The Transcripts from mycoparasitic *Pythium* CBEL proteins, predicted to contain either a TGase_elicitor or an Elicitin domain, were not highly expressed during the interaction with *Ph. infestans* under the conditions tested (Figure 6B).

**FIGURE 10:**
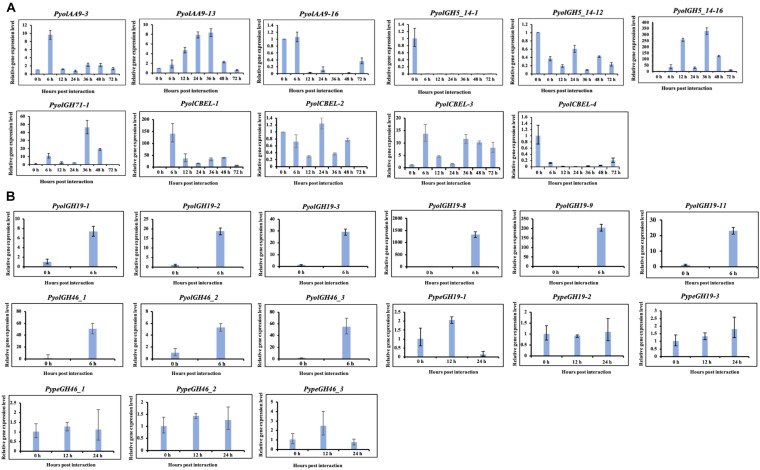
Quantitative Real-Time PCR verification of expression of selected CAZy genes in *P. oligandrum* and *P. periplocum* during *in vitro* growth or during interactions with *Ph. infestans* or *B- cinnerea*. *In vitro* growth at 0 h was used as the reference and normalized to 1.0. The α-tubulin genes of *P. oligandrum* and *P. periplocum* (Pyoltua and Pypetua) were used as internal reference genes. Data displayed as the average of three biological replicates and error bars indicate standard deviations. 6, 12, 24, 36, 48, 72 h represent hours post interaction with *Ph. infestans* or *B. cinerea*. **(A)** Quantitative Real-Time PCR verification for CAZy genes of *P. oligandrum* and *P. periplocum* during interactions with *Ph. infestans.* **(B)** Quantitative Real-Time PCR verification for CAZy genes of *P. oligandrum* and *P. periplocum* during interactions with *B. cinerea*.

Based on qRT-PCR (Figure 10A), transcripts of *PyolCBEL-1*, *PyolCBEL-2*, and *PyolCBEL-3* were all more abundant in the interaction with *Ph. infestans* than during *in vitro* growth of *P. oligandrum*, with *PyolCBEL-1* and *PyolCBEL-3* showing the highest expression levels. *PyolCBEL-1* peaked at almost 150-fold the *in vitro* expression level at 6 hpi compared and *PyolCBEL-3* shows a 12-fold expression increase at the same time point. *PyolCBEL-2* was expressed at near *in vitro* levels throughout the infection time course, and *PyolCBEL-4* was expressed at extremely low levels throughout the interaction. Three genes containing CMB63 domains from *P. periplocum* were highly upregulated during the early stages of the interactions with *Ph. infestans* (Supplementary Figure S2B), but interestingly CBM63 genes were not differentially expressed in the *P. oligandrum-Ph. infestans* interaction. The CBM63 module from *Bacillus subtilis* expansin protein EXLX1 has been experimentally shown to bind cellulose (Nikolaos et al., 2011).

Overall, we compared our RNA-seq data and qRT-PCR assay results (Figures 2B, 3B, 5B, 6B,C, 7B,C, 10 and Supplementary Figure S6). From the RNA-seq analysis we concluded that, *PyolAA9-3*, *PyolAA9-13*, *PyolGH5_14-16*, *PypeGH46-1*, and *PyolCBEL-1* were upregulated during interactions with *Ph. infestans* or *B. cinema*. *PyolAA9-16*, *PyolGH5_14-1*, *PyolCBEL-2*, and *PyolCBEL-4* were downregulated during interaction with *Ph. infestans.* All the above genes mentioned also show similar expression profiles using qRT-PCR assays. *PyolGH5_14-12*, *PypeGH19-1*, *PypeGH19-2*, *PypeGH19-3*, *PypeGH46-2*, *PypeGH46-3*, *PyolCBEL-3* were constitutively expressed. There was generally a good agreement between the RNA-seq and qRT-PCR expression data, showing the reliability of our data. However, *PyolGH5_14-12*, *PypeGH19-1*, and *PyolCBEL-3* expression using RNA-seq is not so consistent with that shown by qRT-PCR.

### Utilization of Complex Carbohydrate Sources

**FIGURE 11:**
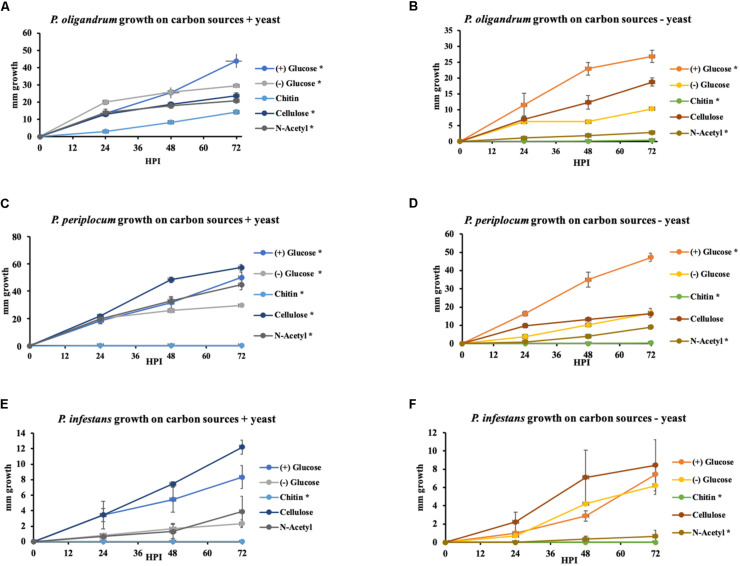
Growth of *P. oligandrum*, *P. periplocum*, and *Ph. infestants* on varying carbon sources representing the major structural carbohydrates in fungal or oomycete cell walls. *P. oligandrum*, *P. periplocum*, and *Ph. infestans* growth on the carbon sources, cellulose, glucose, chitin, and N-Acetyl-glucosamine in modified Plich media plus or minus yeast, denoted by different colored lines. *X*-axis shows hours post inoculation (hpi). *Y*-axis displays colony diameter in mm. **(A,B)** Growth rate of *P. oligandrum*. **(C,D)** Growth rate of *P. periplocum*. **(E,F)** Growth rate of *Ph. infestans*. * denotes statistically significant difference from the control (-glucose, -yeast) data at *p* < 0.05 (*t*-test).

To investigate the extent to which the mycoparasitic oomycetes could directly utilize the major complex carbohydrate components of oomycete and fungal cell walls as the sole sources of carbon we performed *in vitro* growth assays in minimal media amended with either cellulose, chitin or the monomeric unit of chitin, N-acetylglucosamine with glucose or yeast extract amendments used as control conditions. We compared the growth of *P. oligandrum* and *P. periplocum* to that of *Ph. infestans* (Figure 11).

In either the presence or absence of yeast, *P. oligandrum* grew largest with glucose as a carbon source and was also able to utilize cellulose, chitin and N-acetylglucosamine as carbon sources. In the presence of yeast, *P. periplocum* grew equally well in most treatments, but in the absence of yeast, grew best with glucose as the sole carbon source. In the absence of yeast *P. periplocum* showed a strong preference for growth in glucose media and very reduced growth in the other substrates. In either the presence or absence of yeast, *P. infestans* grew largest with cellulose as the primary carbon source, and was not able to utilize chitin or N-acetylglucosamine reflecting the adaptation of this species to cellulosic plant hosts. Several CAZy encoded genes that function in the release of the simple sugar glucose from cellulose (e.g., GH6 and GH17 family members) were upregulated during mycoparasitism of the cellulosic host *Ph. infestans*, and genes for the degradation of the cell wall carbohydrates from fungal and oomycete prey were abundant and expanded in the mycoparasite genomes.

Taken together, these data suggest that degradation of more complex carbohydrate constituents of fungal and oomycete cell walls is only of limited importance to mycoparasitic oomycetes. Instead, destruction of the major components of prey cell walls in order to gain access to simple sugars within the cells appears to be the predominant strategy of these mycoparasites. This is in line with similar strategies used by phytopathogenic *Pythium* species, where CWDE secretion to allow maceration of the plant cell wall and subsequent uptake of simple sugars from within plant cells appears to be the major mechanism by which these species obtain nutrition (Zerillo et al., 2013). However, due to the expansion of CWDEs within mycoparasitic oomycete genomes they show a clear ability to utilize more complex carbohydrates not found within the plant cell wall when necessary. The mycoparasites also exhibit overall faster growth *in vitro* than the plant pathogen indicating a more efficient usage of these carbon sources for growth, and potentially allowing these species to outcompete their plant pathogenic or saprotrophic counterparts within the agroecosystem.

## Conclusion

*P. oligandrum* and *P. periplocum* have long been recognized for their ability to inhibit plant pathogens (Paul, 1999; Rey et al., 2008), although the molecular basis of their interactions remains largely unknown. A small cDNA library sequencing study previously identified the potential role of CWDEs in *P. oligandrum* mycoparasitism (Horner et al., 2012). Through a detailed *in silico* and transcriptomic analysis, we have investigated the CAZyome of both *P. oligandrum* and *P. periplocum* in relation to plant pathogenic oomycetes. In conclusion, we found that tandem duplication and horizontal gene transfer events have been the major drivers for the formation of the distinctive and expanded CAZyome of mycoparasitic *Pythium* species.

We identified three major CAZy families from which the member genes are predominantly located in the gene sparse regions of their respective genomes and that have expanded through tandem duplication. These are hallmarks of genes undergoing rapid evolution such as the RxLR effectors of plant pathogenic oomycetes (Haas et al., 2009; Dong et al., 2015). Based on our data, we postulate, for the first time, that mycoparasitic oomycete genomes also display a “two-speed genome” phenomenon in a similar manner to plant pathogenic oomycetes, but that the key pathogenicity determinants, at least those responsible for the onset of infection are CAZyme CWDEs that afford the mycoparasitic oomycetes the opportunity to macerate microbial tissue from which they can extract the sugars and other nutrients they require for growth and reproduction.

We have identified that carbohydrate active enzymes such as cellulose degrading enzymes are potentially important pathogenicity determinates in the mycoparasitic oomycetes. Since both mycoparasite and prey oomycetes all contain such enzymes, we hypothesize that these enzymes must be tightly regulated or compartmentalized to limit or prevent self-degradation and thus the regulation of CAZy gene function in mycoparasitic oomycetes is an important topic for further investigation in the future. Such studies will help to answer one of the most important questions in mycoparasitism; how self, and non-self, recognition occurs.

Furthermore, we have provided evidence for the horizontal gene transfer of five CAZy families from bacteria, fungi or viruses to the mycoparasites. In contrast, mycoparasitic fungi of the *Trichoderma* species are reported to have obtained almost 50% of their CWDE complement by horizontal, or lateral, gene transfer (Druzhinina et al., 2018). However, Richards et al. (2011) investigated the occurrence of horizontal gene transfer events from fungi to oomycetes and concluded that only a small number of such events could be found, thus our results are in line with these previous findings (Richards et al., 2011).

Taken together our data suggest a possible phytopathogenic ancestral state for the *Pythium* Genus, with the ability to mycoparasitise other eukaryotic microbes an adaptation derived from expansion of several key CAZyme gene families, and horizontal gene transfer events that occurred in the last common ancestor of *P. oligandrum* and *P. periplocum*.

## Materials and Methods

### Isolates and Sequence Retrieval for Comparative CAZyme Analysis

The published genomes of the following oomycetes were used for the comparative analysis of their CAZyme encoding gene complement: *P. oligandrum* (CBS 530.74) (Kushwaha et al., 2017a), *P. periplocum* (CBS 532.74) (Kushwaha et al., 2017b), *P. ultimum* (DAOM BR144) (Lévesque et al., 2010), *P. irregulare* (DAOM BR486) (Adhikari et al., 2013), *P. iwayamai* (DAOM BR242034) (Adhikari et al., 2013), *P. arrhenomanes* (ATCC 12531) (Adhikari et al., 2013), *P. aphanidermatum* (DAOM BR444) (Adhikari et al., 2013), *P. insidiosum* (Strain Pi-S) (Rujirawat et al., 2015), *Phytopythium. vexans* (DAOM BR484) (Adhikari et al., 2013), *Ph. infestans* (T30-4) (Haas et al., 2009), *Ph. sojae* (P6497) (Tyler et al., 2006), *Ph. ramorum* (Pr102) (Tyler et al., 2006), *Ph. capsici* (LT1534) (Lamour et al., 2012).

To explore overall distribution of CAZymes among the above 11 oomycetes, the genome assemblies, transcript sequences, and protein sequences were downloaded from the NCBI Genome portal^1^ and the Joint Genome Portal^2^, along with the gene model annotation files in GFF3 format, which were used for CAZy annotation and synteny analysis.

### BUSCO Comparisons

Benchmarking Universal Single-Copy Orthologs BUSCO v4 (Seppey et al., 2019)^3^ was used to evaluate the completeness of the genome assemblies of the oomycetes used in this study. Default parameters were used with lineage-specific datasets set to stramenopiles_odb10.

### Growth and Maintenance of Oomycete and Fungal Species

*P. oligandrum* (CBS 530.74) and *P. periplocum* (CBS 532.74) were maintained on V8 media amended with CaCO_3_ as previously described (Horner et al., 2012; Kushwaha et al., 2017a, b) at 18°C in the dark. *Ph. infestans* (88,069) was maintained on rye sucrose agar at 18°C in the dark as described (Grenville-Briggs et al., 2008). *B. cinerea* (B05) was maintained on either V8 media or corn meal agar (CMA) at 20°C in the dark. Prior to confrontation, *P. oligandrum*, *P. periplocum* and *B. cinnerea* were grown in liquid V8 broth amended with calcium carbonate and *Ph. infestans* were grown in liquid pea broth, at 20°C in the dark.

### Annotation of the CAZyome

CAZyme-encoding genes were predicted using the dbCAN pipeline (Yin et al., 2012). Briefly, hidden Markov models of all CAZy families were download from CAZy database (Lombard et al., 2013)^4^. Hidden Markov searches using the predicted proteomes of the oomycete species listed above were performed, with a cut-off value of 1E-03. To predict members of the CBEL family, only candidates containing two CBM_1 domain and a signal peptide were retained.

Transmembrane domain searches were conducted using the TMHMM Server v2.0 (Käll et al., 2005)^5^. SignalP 3.0 was used to predict the presence of a signal peptide for secretion (Bendtsen et al., 2004)^6^. Domain architecture predictions were conducted with by searching the Pfam database (El-Gebali et al., 2018)^7^. For CAZy families with diverse enzyme activity, the exact enzyme activity annotation of members in these families were conducted by using BLASTP search of verified proteins in the ExPASy database (Artimo et al., 2012)^8^.

### Identification of CAZy Families Specific to, or Expanding in, the Mycoparasitic *Pythiums*

Fisher’s exact test, conducted in the R program suite v3.5.3, was used to compare the gene counts of CAZy family members in the mycoparasitic *Pythium* species with those of the plant pathogenic *Pythium*, human *Pythium*, and *Phytophthora* species, respectively. The CAZy families with *p*-values higher than 1E-05 were removed. The CAZy families with higher gene counts in mycoparasitic *Pythium* were thus retained for further analysis.

### Phylogenetic Analysis

Protein sequences were aligned by MUSCLE (Edgar, 2004). Phylogenetic analysis of full-length proteins alignments was performed by IQ-TREE (Nguyen et al., 2014), using the Maximum Likelihood approach and 1,000 bootstrap values. The models for phylogenetic analysis were automatically selected by IQ-TREE program. The visualization and modification of phylogenetic trees were performed using the iTOL server (Letunic and Bork, 2006). Branches with the bootstrap values higher than 50 were displayed.

### Synteny Analysis

The predicted proteomes of *P. oligandrum* and *P. periplocum* were used to search against the predicted proteome of *P. ultimum* using BLASTP, using the default maximum hits setting with an *E*-value cut-off of 1E-10. Identification of gene collinearity between mycoparasitic *Pythium* and *P. ultimum* was performed using MCScanX (Wang et al., 2012). TBtools (Chen et al., 2018) was employed for visualization of the synteny analysis.

### Evaluation of Average Fold Change of CAZy Families

We screened significantly differentially expressed CAZy genes and the CAZy families they belong to. By calculating the average fold change of these CAZy families, with treatment by log2, we could distinguish overall expression profiles of differentially expressed CAZy genes that were assigned to the same CAZy family. We used a log2-treated average fold change approach, to analyze the changes in expression of genes from each family where values equal to, or less than, −1 (to the left of the dashed black line in Supplementary Figure S2) correspond to down regulation of most of the genes within a family and values equal to, or greater than, 1 (to the right of the dashed red line in Supplementary Figure S2) correspond to up-regulation of the majority of the genes in that family. Numbers of genes from each gene family, on which this analysis is based are shown in the left of each panel of Supplementary Figure S2.

### Mycoparasite-Prey Confrontation Assays

For confrontation assays, approximately 5 cm^3^ of mycelium, washed with sterile dH_2_0, from mycoparasite (*P. oligandrum* or *P. periplocum*) and prey (either *Ph. infestans* or *B. cinnerea*) were placed at opposite sides of a polycarbonate membrane on V8 agar. The interaction zone (1 cm) was sampled at the point of contact (0 h post interaction, hpi) and then subsequently at 12 and 24 hpi. Control samples of the mycoparasites were prepared using two mycelial plugs from the same organism interacting with each other for 3 days, and mycoparasitism samples were prepared using either the oomycete *Ph. infestans* or the fungus *B. cinnerea* as the prey, which was prepared from liquid media samples as described above. Collected samples were snap frozen in liquid nitrogen and used for RNA extraction. Five replicates of each interaction were prepared and three of these replicates were randomly chosen for RNA extraction and sequencing. For detailed quantitative RT-PCR of a time course of mycoparasitism, the interacting mycelium from the confrontations between the mycoparasite and the prey in sterile tap water, was excised at 0, 6, 12, 24, 36, 48, and 72 hpi and immediately snap frozen and ground in liquid nitrogen, prior to RNA extraction.

### RNA Extraction

Approximately 100 mg RNA from each sample was extracted using the RNeasy Plant Mini Kit (#74904 QIAGEN) according to the manufacturers protocol, and treated with RNase-free DNase for 20 min at 37°C (Ambion, TURBO DNA-free Kit). The extracted RNA was qualitatively visualized by agarose gel electrophoresis and a NanoDrop 1,000 spectrophotometer (Thermo Fisher Scientific) was used to quantify the total amount of RNA. Prior to RNA sequencing, the integrity of the samples was corroborated using the Experion^TM^ Automated Electrophoresis System (Bio-Rad Laboratories, Hercules, United States).

### Expression Analysis From RNA Sequencing

Polyadenylated messenger RNA was captured from 200 ng total RNA per sample using magnetic beads and Illumina adaptors with sample specific barcode sequences were ligated before subsequent library amplification using PCR using the Illumina TruSeq RNA poly-A selection kit. Sequencing of 150 bp paired-end libraries was carried out using the Illumina NovaSeq6000 S4 platform (SciLifeLab, Stockholm). All raw sequencing data in this study have been deposited in National Center for Biotechnology Information (NCBI) under BioProject accession number PRJNA637834 and a full analysis of these data will be presented elsewhere. The resulting bcl2fastq demultiplexed FastQ files were *de novo* mapped to our previously published reference genomes (Kushwaha et al., 2017a, b) and Adaptor cleaning was conducted by Trimmomatic (Bolger et al., 2014). Normalization and quantification of expression levels was performed by Salmon (Patro et al., 2015). The full analysis of differential gene expression during mycoparasitic interactions will be published elsewhere, however, in the current study we have mined these data to investigate the expression levels of selected CAZyme-encoding genes as presented in the results. pheatmap package of R project (Kolde, 2012) was used to produce heatmaps showing the log fold change in gene expression values (TPM counts) for selected CAZyme-encoding genes between the transcriptome samples.

### Validation of Gene Expression Using Quantitative RT-PCR

cDNA synthesis was carried out using the Superscript IV Reverse transcriptase cDNA synthesis kit (Thermo Fisher Scientific) using 2 μg template RNA. All cDNA samples were diluted to 20 ng^–1^ prior to qRT-PCR. The gene expression levels were evaluated using quantitative RT-PCR (Biorad real-time PCR cycler using SYBG as the fluorescent dye). *P. oligandrum* and *P. periplocum* α-tubulin genes (*Pyoltua*; Genbank accessions MT623563 and MT811915; Supplementary Table S1). Primers (listed in Supplementary Table S1) were designed in Primer3^9^ and the NCBI BLASTN web platform was used to check the specificity of the sequences for the genes in question, with the low complexity filter turned off. The internal reference genes list above were used to normalize expression levels of CAZyme candidates from the corresponding species.

### Growth Assays to Investigate Utilization of Complex Carbohydrate Carbon Sources

To study growth of mycoparasitic *Pythium* on different carbon substrates, representing the major structural components of oomycete and fungal cell walls, we compared the growth of *P. oligandrum, P. periplocum*, and *Ph. infestans* in modified Plich media (van West et al., 1999) amended with, cellulose, glucose, chitin and N-Acetylglucosamine individually at 25 mM^∗^mL^–1^ both with and without yeast extract. Modified plich media without glucose or yeast extract served as controls. 7.5 mm plugs from 7-day old liquid cultures of *P. oligandrum* CBS 530.74 and *P. periplocum* CBS 532.74 cultivated in V8 media and *Ph. infestans* 88,069 cultivated in pea-broth were used to investigate the growth rate of the oomycetes on different the carbon sources. *Ph. infestans* was used as a comparative control. Growth of each organism was quantified as diagonal growth in mm and measured every 24 hpi for three constitutive days. In total 10 treatments were performed. The experiment was repeated three times with three independent biological replicates. Statistical analysis of the growth assays was calculated as the area under the growth curve using the trapezoidal method. The differences among treatments was assessed using student’s *T*-test, on the means of the area under the curve, assuming two-tailed distribution and two-sample with unequal variance. Significant difference was accepted if (*p* < 0.05).

## Data Availability Statement

RNA-seq data (NCBI accession: PRJNA637834); *P. oligandrum* internal reference gene (Genbank accession: MT623563); *P. periplocum* internal reference gene (Genbank accession: MT811915).

## Author Contributions

LG-B designed the experiments and analysis methods. DL designed and performed the bioinformatic analysis. CA and RV designed and performed the wet lab experiments and analyzed the wet lab experimental data. DL, CA, and LG-B wrote the manuscript. LG-B and DD revised the manuscript. All authors reviewed and approved the final manuscript.

## Conflict of Interest

The authors declare that the research was conducted in the absence of any commercial or financial relationships that could be construed as a potential conflict of interest.

## Footnotes

1 https://www.ncbi.nlm.nih.gov/genome

2 https://mycocosm.jgi.doe.gov/mycocosm/home

3 www-busco.ezlab.org

4 http://www.cazy.org/

5 http://www.cbs.dtu.dk/services/TMHMM/

6 http://www.cbs.dtu.dk/services/SignalP-3.0/

7 https://pfam.xfam.org/

8 https://www.expasy.org/

9 http://primer3.wi.mit.edu

## Supplementary Material

Supplementary Figure 1 BUSCO analysis for evaluation of genome assembly completeness. *X*-axis represent genome assemblies of organisms mentioned in this article. *Y*-axis represent number of each category of BUSCO groups.

Supplementary Figure 2 Comparison of significantly expressed genes assigned to CAZy families that are not unique or expanded in the mycoparasitic *Pythium* species. Numbers within the heatmaps represent the count of significantly expressed genes assigned to each corresponding CAZy family. Green histograms represent log_2_-treated average fold change of Pythium CAZy genes significantly expressed from *in vitro* growth to 12 hpi in the presence of *Ph. infestans*. Light blue histograms represent log_2_-treated average fold change of *Pythium* CAZy genes significantly expressed from 12 hpi in the presence of *Ph. infestans* to 24 hpi in the same interaction. Black and red dashed lines represent log_2_-treated average fold change equal to -1 and 1 respectively. **(A)** overview of significantly expressed CAZy genes of *P. oligandrum*. **(B)** overview of significantly expressed CAZy genes of *P. periplocum*.

Supplementary Figure 3 Analysis of gene density for selected CAZy gene families. **(A)** Gene density heatmap of AA9 genes in *P. oligandrum.* **(B)** Gene density heatmap of GH5_14 genes in *P. oligandrum*. Dots with different color represent intergenic distance of encoding genes in different clades according to respective phylogenic tree. **(C)** Gene density heatmap of GH19 genes in *P. oligandrum*. **(D)** Gene density heatmap of GH19 genes in *P. periplocum*. Dots with black color represent intergenic distance of GH19 encoding genes detected in *P. oligandrum* and *P. periplocum*.

Supplementary Figure 4 Analysis of genome location of selected CAZy genes. **(A)** Genome location of *P. oligandrum* AA9 encoding genes from clades 1 and 3. Blue arrows represent the orientation of *P. oligandrum* AA9 encoding genes from clades 1 and 3. **(B)** Genome location of *P. oligandrum* GH5_14 encoding genes encoding from clade 1. Blue arrows represent the orientation of *P. oligandrum* GH5_14 genes from clade 1. **(C)** Genome location of *P. oligandrum* GH19 genes. Blue arrows represent orientation of *P. oligandrum* GH19 genes.

Supplementary Figure 5 Synteny analysis. **(A)** Synteny analysis of GH55 genes between mycoparasitic *Pythium* species and *P. ultimum*. The red arrows represent GH55 genes identified in *P. oligandrum* and *P. periplocum*. The blue arrows represent genes near to GH55 genes. The gray lines represent syntenic relationships. **(B)** Synteny analysis of GH46 genes between mycoparasitic *Pythium* and *P. ultimum*. The red arrows represent the orientation of GH46 genes identified in *P. oligandrum* and *P. periplocum*. The blue arrows represent the orientation of genes neighboring the GH46 genes. The gray lines represent syntenic relationships.

Supplementary Figure 6 Analysis of the cellulose-binding elicitor lectin (CBEL) family. **(A)** Phylogenetic tree of CBEL proteins identified in oomycete species. Maximum likelihood tree, with 1,000 bootstraps (values displayed per branch). CBEL proteins identified in *P. oligandrum* (Pyol) and *P. periplocum* (Pype) are marked in red. TrCBHI_P62694 (an exoglucanase verified in *Trichoderma reesei*) was used as an outgroup. Domain architecture of CBEL proteins is shown on to the left of the phylogenetic tree. **(B)** RNAseq Expression profile of *CBEL* genes detected in *P. oligandrum* and *P. periplocum* during *in vitro* growth or during interactions with *Ph. infestans* at 12 or 24 h post interaction. Expression levels are expressed as the log_2_ fold change of transcripts per million (TPM), per gene.

Supplementary Figure 7 Analysis of the cellulose synthase (CesA) family. **(A)** Phylogenetic tree of CesA proteins identified in oomycete species. Maximum likelihood tree, with 1,000 bootstraps (values displayed per branch). CesA3 proteins identified in *P. oligandrum* (Pyol) and *P. periplocum* (Pype) are marked in red. CesA proteins identified in *Arabidopsis thaliana* (At) were used as outgroup genes. The domain architecture of each of the CesA proteins is shown to the left of the Phylogenic tree. **(B)** RNAseq Expression profile of CesA genes detected in *P. oligandrum* and *P. periplocum* during *in vitro* growth or during interactions with *Ph. infestans* at 12 or 24 h post interaction. Expression levels are expressed as the log_2_ fold change of transcripts per million (TPM), per gene.

Supplementary Figure 8 Phylogenic analysis of GH55 and GH71 proteins detected in mycoparasite *Pythium* and their homologous proteins detected by BLAST search. Maximum likelihood tree, with 1,000 bootstraps (values displayed per branch). Proteins identified in *P. oligandrum* (Pyol) and *P. periplocum* (Pype) are shown in red.

Supplementary Table 1 Primers for CAZyme-coding genes verified by qRT-PCR in this study.

Supplementary Table 2 ID and gene model sequences of CAZyme-coding genes in this study.

Supplementary Table 3 Ortholog clustering for each CAZy families mentioned in this study.
