# Supplementary material for: Horizontal Gene Transfer and Tandem Duplication Shape the Unique CAZyme Complement of the Mycoparasitic Oomycetes Pythium oligandrum and Pythium periplocum
Source: Front Microbiol. 2020 Oct 29;11:581698. doi: 10.3389/fmicb.2020.581698 (PMC7720654; doi:10.3389/fmicb.2020.581698)
Supplement: Supplementary Figure 1 — BUSCO analysis for evaluation of genome assembly completeness. X-axis represent genome assemblies of organisms mentioned in this article. Y-axis represent number of each category of BUSCO groups. [file Data_Sheet_1.PDF]

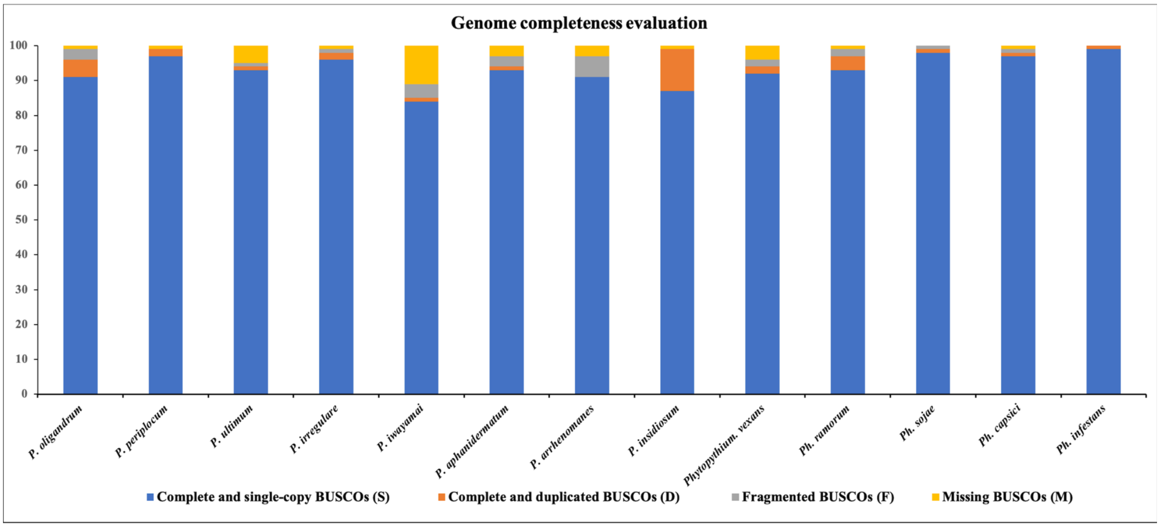

Fig S1. BUSCO analysis for evaluation of genome assembly completeness. X-axis represent genome assemblies of organisms mentioned in this article. Y-axis represent number of each category of BUSCO groups.

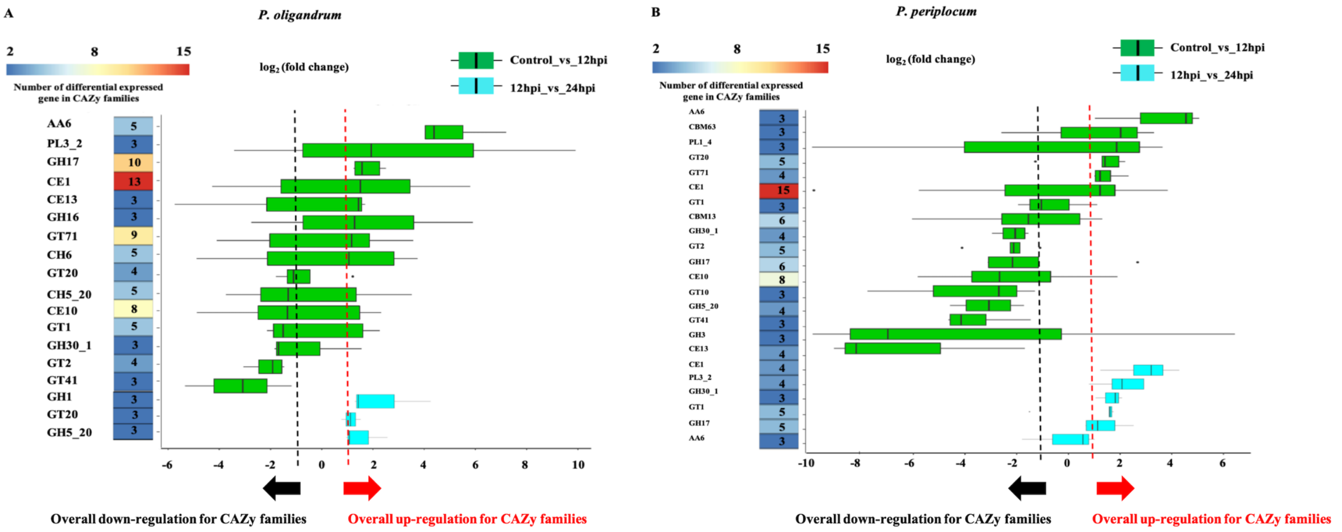

Fig S2. Comparison of significantly expressed genes assigned to CAZy families that are not unique or expanded in the mycoparasitic *Pythium* species. Numbers within the heatmaps represent the count of significantly expressed genes assigned to each corresponding CAZy family. Green histograms represent $\log_2$-treated average fold change of Pythium CAZy genes significantly expressed from *in vitro* growth to 12 hpi in the presence of *Ph. infestans*. Light blue histograms represent $\log_2$-treated average fold change of *Pythium* CAZy genes significantly expressed from 12 hpi in the presence of *Ph. infestans* to 24 hpi in the same interaction. Black and red dashed lines represent $\log_2$-treated average fold change equal to -1 and 1 respectively. **(A) overview of significantly expressed CAZy genes of *P. oligandrum*. (B) overview of significantly expressed CAZy genes of *P. periplocum*.**

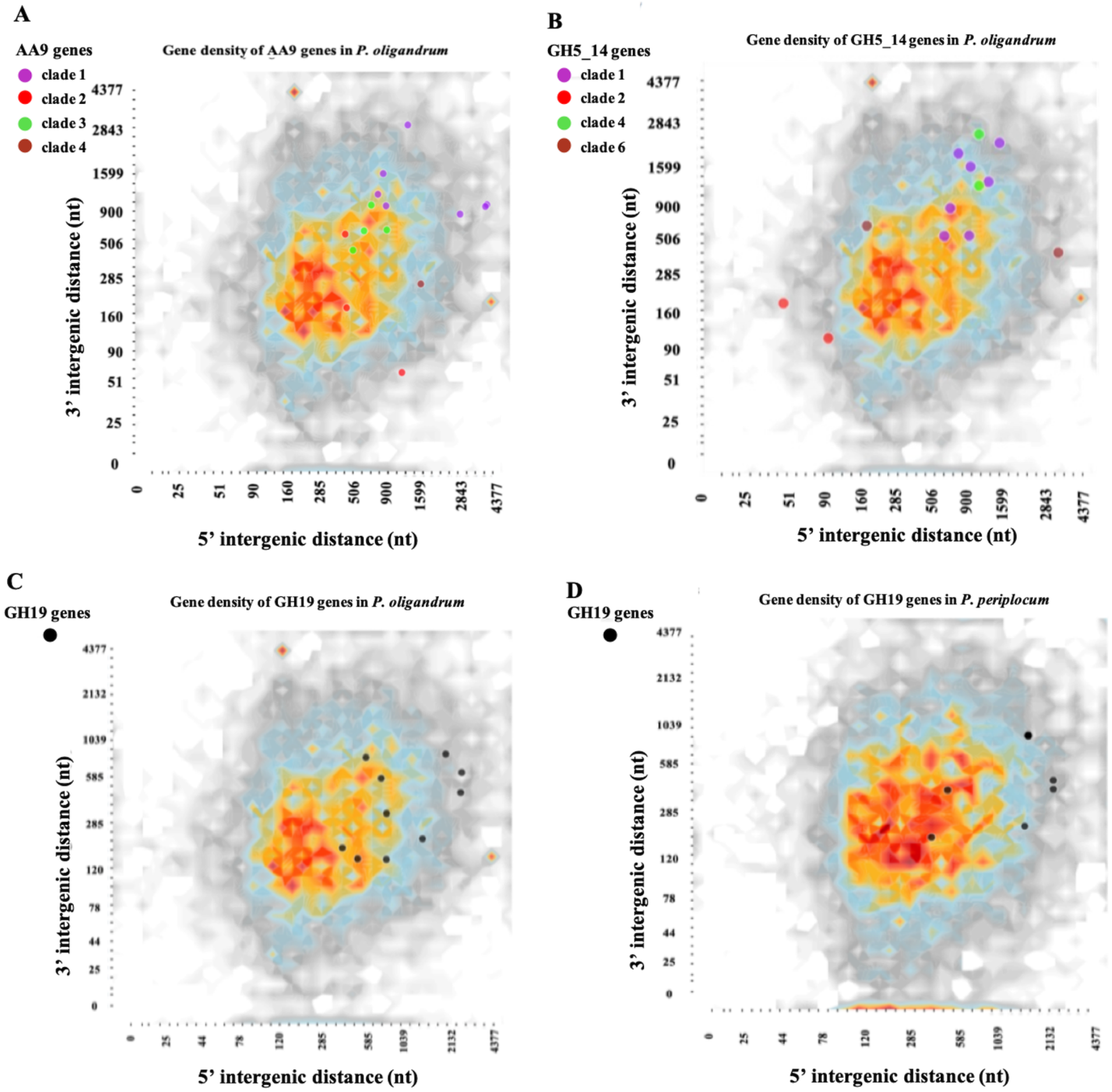

Fig S3. Analysis of gene density for selected CAZy gene families. (A) Gene density heatmap of AA9 genes in *P. oligandrum.* (B) Gene density heatmap of GH5_14 genes in *P. oligandrum*. Dots with different color represent intergenic distance of encoding genes in different clades according to respective phylogenic tree.

(C) Gene density heatmap of GH19 genes in *P. oligandrum*. (D)Gene density heatmap of GH19 genes in *P. periplocum*. Dots with black color represent intergenic distance of GH19 encoding genes detected in *P. oligandrum* and *P. periplocum*.

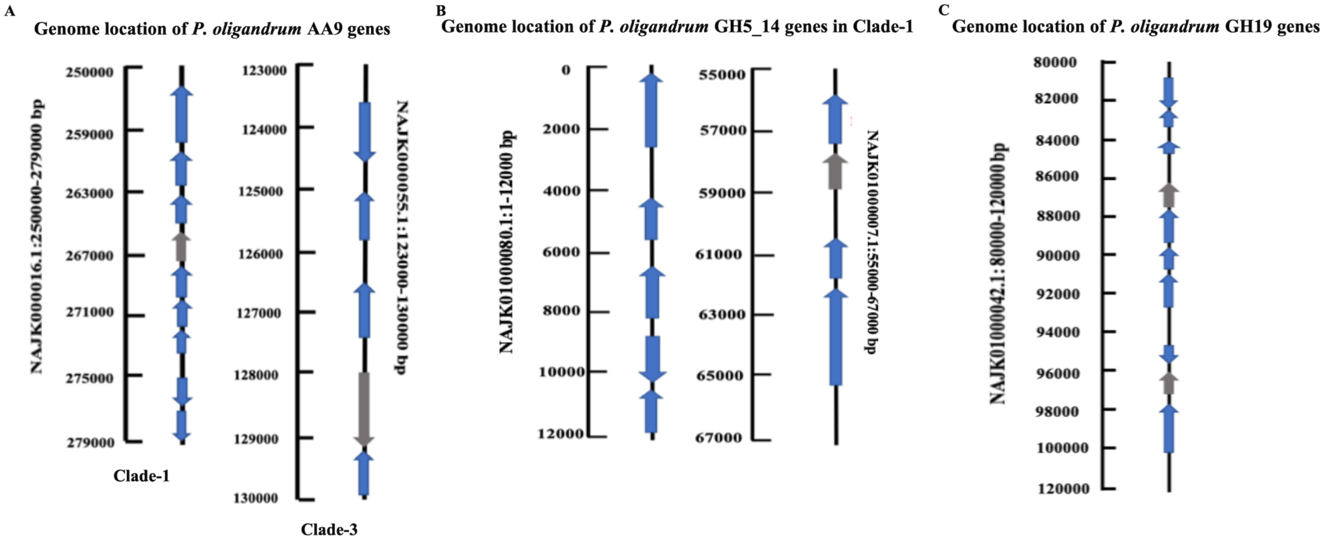

Fig S4. Analysis of genome location of selected CAZy genes. (A) Genome location of *P. oligandrum* AA9 encoding genes from clades 1 and 3. Blue arrows represent the orientation of *P. oligandrum* AA9 encoding genes from clades 1 and 3. **(B) Genome location of *P. oligandrum* GH5_14 encoding genes encoding from clade 1.** Blue arrows represent the orientation of *P. oligandrum* GH5_14 genes from clade 1. **(C) Genome location of *P. oligandrum* GH19 genes.** Blue arrows represent orientation of *P. oligandrum* GH19 genes.

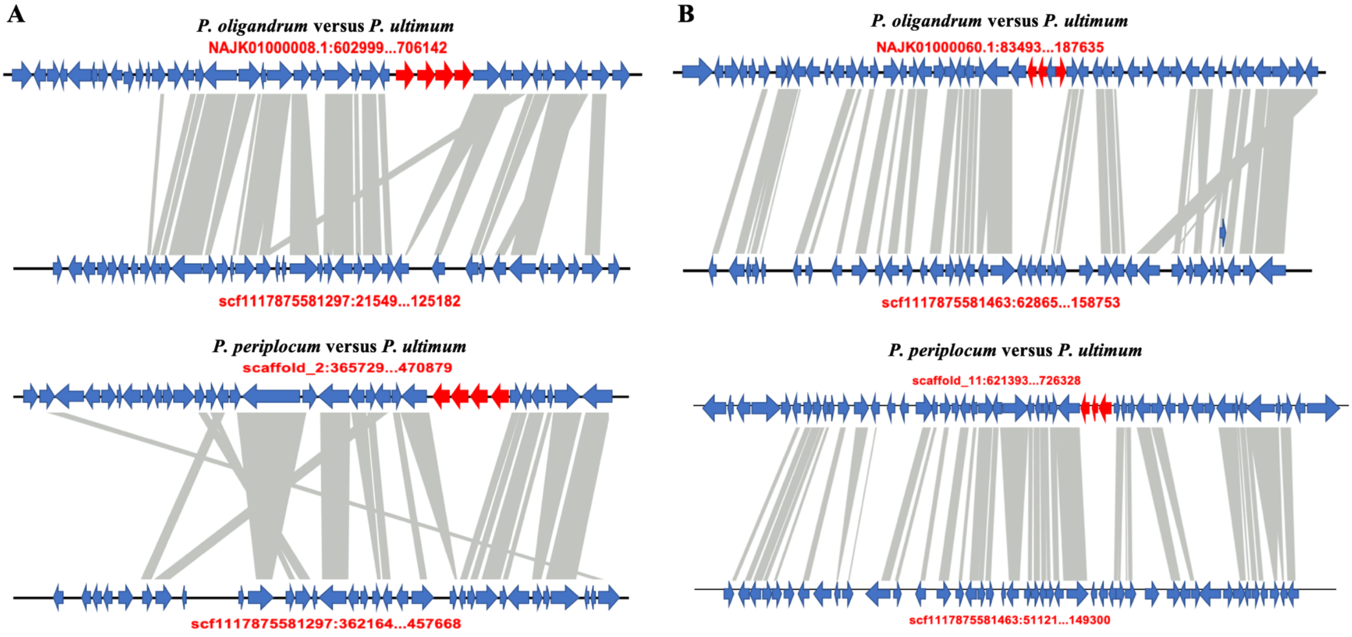

Fig S5. Synteny analysis. (A) Synteny analysis of GH55 genes between mycoparasitic *Pythium* species and *P. ultimum*. The red arrows represent GH55 genes identified in *P. oligandrum* and *P. periplocum*. The blue arrows represent genes near to GH55 genes. The grey lines represent syntenic relationships. **(B) Synteny analysis of GH46 genes between mycoparasitic *Pythium* and *P. ultimum*.** The red arrows represent the orientation of GH46 genes identified in *P. oligandrum* and *P. periplocum*. The blue arrows represent the orientation of genes neighboring the GH46 genes. The grey lines represent syntenic relationships.

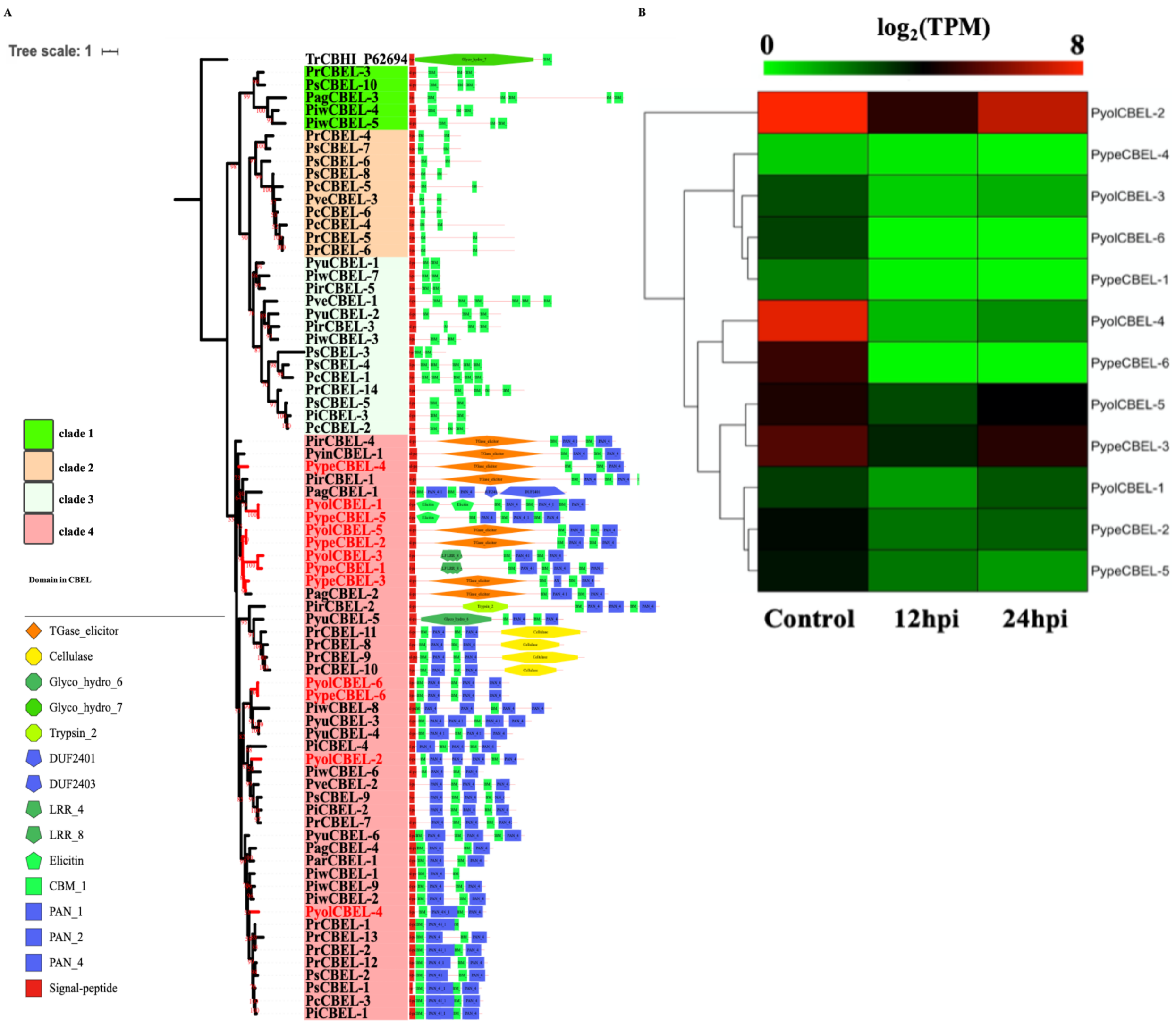

Fig S6. Analysis of the cellulose-binding elicitor lectin (CBEL) family. (A) Phylogenetic tree of CBEL proteins identified in oomycete species. Maximum likelihood tree, with 1000 bootstraps (values displayed per branch). CBEL proteins identified in *P. oligandrum* (Pyol) and *P. periplocum* (Pype) are marked in red. TrCBHI_P62694 (an exoglucanase verified in *Trichoderma reesei*) was used as an outgroup. Domain architecture of CBEL proteins is shown on to the left of the phylogenetic tree. **(B) RNAseq Expression profile of *CBEL* genes detected in *P. oligandrum* and *P. periplocum* during *in vitro* growth or during interactions with *Ph. infestans* at 12 or 24-hours post interaction.** Expression levels are expressed as the log_2 fold change of transcripts per million (TPM), per gene.

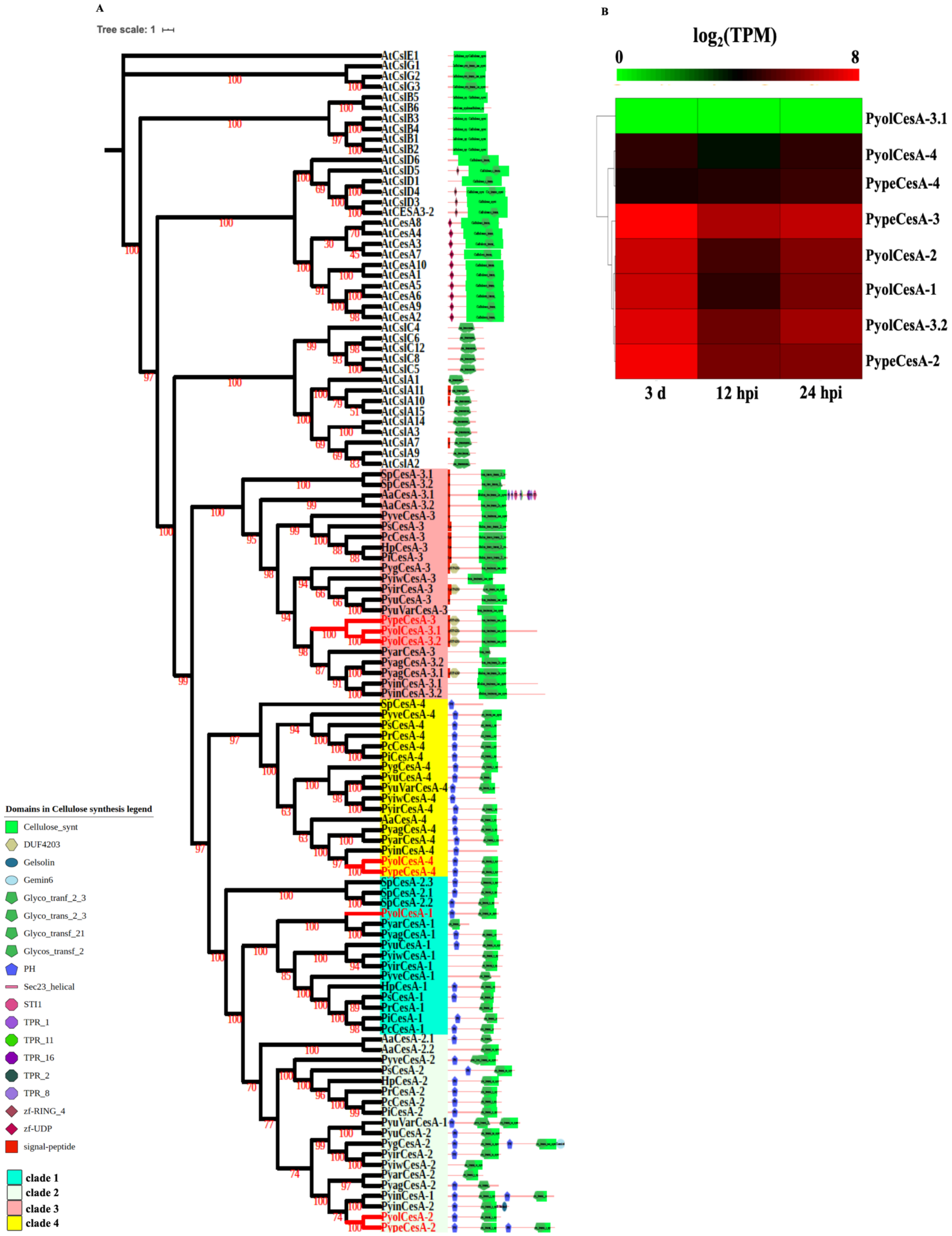

Fig S7. Analysis of the cellulose synthase (CesA) family. (A) Phylogenetic tree of CesA proteins identified in oomycete species. Maximum likelihood tree, with 1000 bootstraps (values displayed per branch). CesA3 proteins identified in *P. oligandrum* (Pyol) and *P. periplocum* (Pype) are marked in red. CesA proteins identified in *Arabidopsis thaliana* (At) were used as outgroup genes. The domain architecture of each of the CesA proteins is shown to the left of the Phylogenic tree. **(B) RNAseq Expression profile of CesA genes detected in *P. oligandrum* and *P. periplocum* during *in vitro* growth or during interactions with *Ph. infestans* at 12 or 24-hours post interaction.** Expression levels are expressed as the $\log_2$ fold change of transcripts per million (TPM), per gene.

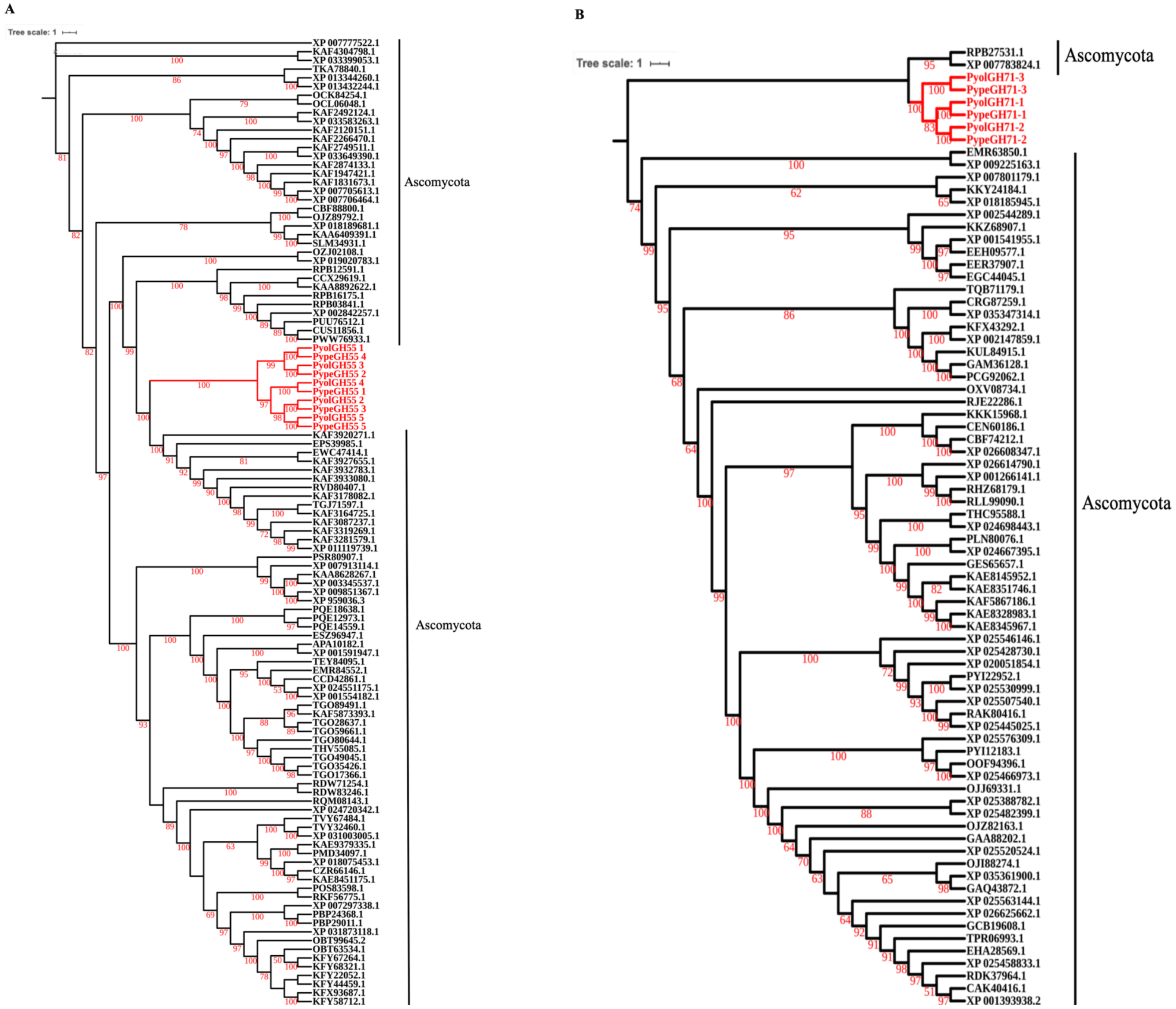

Fig S8. Phylogenic analysis of GH55 and GH71 proteins detected in mycoparasite *Pythium* and their homologous proteins detected by BLAST search. Maximum likelihood tree, with 1000 bootstraps (values displayed per branch). Proteins identified in *P. oligandrum* (Pyol) and *P. periplocum* (Pype) are shown in red.
