# Supplementary material for: Horizontal Gene Transfer and Tandem Duplication Shape the Unique CAZyme Complement of the Mycoparasitic Oomycetes Pythium oligandrum and Pythium periplocum
Source: Front Microbiol. 2020 Oct 29;11:581698. doi: 10.3389/fmicb.2020.581698 (PMC7720654; doi:10.3389/fmicb.2020.581698)
Supplement: Supplementary Table 2 — ID and gene model sequences of CAZyme-coding genes in this study. [file Table_3.pdf]

AA9 family	OG0000000	*PagAA9-2 PagAA9-3 PagAA9-4 ParAA9-1 ParAA9-3 PsAA9-1 PsAA9-2 PsAA9-3 PveAA9-1 PyinAA9-1 PyolAA9-1 PyolAA9-10 PyolAA9-11 PyolAA9-12 PyolAA9-13 PyolAA9-14 PyolAA9-15 PyolAA9-16 PyolAA9-2 PyolAA9-4 PyolAA9-6 PyolAA9-7 PyolAA9-8 PyolAA9-9 PypeAA9-1 PypeAA9-10 PypeAA9-12 PypeAA9-13 PypeAA9-14 PypeAA9-15 PypeAA9-16 PypeAA9-17 PypeAA9-3 PypeAA9-4 PypeAA9-6 PypeAA9-7 PypeAA9-8 PypeAA9-9*
	OG0000001	*PagAA9-5 ParAA9-2 ParAA9-4 PcAA9-1 PiwAA9-1 PrAA9-1 PrAA9-2 PyolAA9-3 PyolAA9-5 PypeAA9-5*
	OG0000002	*PagAA9-1 PypeAA9-2*
	OG0000003	*PypeAA9-11*
GH5_14 family	OG0000000	*PcGH5_14-3 PcGH5_14-4 PcGH5_14-5 PiGH5_14-8 PiGH5_14-9 PrGH5_14-1 PrGH5_14-3 PrGH5_14-4 PsGH5_14-4 PsGH5_14-5 PsGH5_14-6 PyarGH5_14-11 PyarGH5_14-12 PyarGH5_14-8 PyinGH5_14-2 PyirGH5_14-1 PyirGH5_14-4 PyiwGH5_14-1 PyiwGH5_14-3 PyiwGH5_14-5 PyolGH5_14-1 PyolGH5_14-13 PyolGH5_14-16 PyolGH5_14-2 PyolGH5_14-4 PypeGH5_14-10 PypeGH5_14-12 PypeGH5_14-13 PypeGH5_14-14 PypeGH5_14-15 PypeGH5_14-3 PypeGH5_14-5 PypeGH5_14-9 PyuGH5_14-3 PyuGH5_14-4 PyuGH5_14-5 PyuGH5_14-8 PyuGH5_14-9 PyuVarGH5_14-2 PyuVarGH5_14-3 PyveGH5_14-1 PyveGH5_14-2 PyveGH5_14-3*
	OG0000001	*PcGH5_14-1 PcGH5_14-2 PrGH5_14-10 PrGH5_14-5 PrGH5_14-6 PrGH5_14-7 PrGH5_14-8 PrGH5_14-9 PsGH5_14-2 PsGH5_14-3 PsGH5_14-7 PsGH5_14-8 PyarGH5_14-1 PyarGH5_14-13 PyarGH5_14-4 PyarGH5_14-5 PyarGH5_14-6 PyarGH5_14-9 PyinGH5_14-3 PyinGH5_14-4 PyinGH5_14-6 PyinGH5_14-8 PyinGH5_14-9 PyirGH5_14-3 PyiwGH5_14-2 PyolGH5_14-10 PyolGH5_14-17 PyolGH5_14-18 PypeGH5_14-4 PypeGH5_14-6 PypeGH5_14-7 PypeGH5_14-8 PyuGH5_14-1 PyuGH5_14-2 PyuVarGH5_14-1 PyuVarGH5_14-4 PyveGH5_14-4 PyveGH5_14-5*
	OG0000002	*PcGH5_14-6 PiGH5_14-7 PrGH5_14-2 PsGH5_14-1 PyagGH5_14-2 PyarGH5_14-7 PyinGH5_14-1 PyirGH5_14-5 PyiwGH5_14-4 PyolGH5_14-5 PypeGH5_14-1 PyuGH5_14-7 PyveGH5_14-6*
	OG0000003	*PyagGH5_14-3 PyagGH5_14-4 PyarGH5_14-3 PyinGH5_14-5 PyinGH5_14-7 PyirGH5_14-2 PyolGH5_14-6 PyolGH5_14-7 PyolGH5_14-8 PyolGH5_14-9 PypeGH5_14-2 PyuGH5_14-6*
	OG0000004	*PyagGH5_14-5 PyolGH5_14-11 PyolGH5_14-12 PyolGH5_14-15*
	OG0000005	*PyarGH5_14-2 PyiwGH5_14-6*
	OG0000006	*PyolGH5_14-3 PypeGH5_14-11*
GH55 family	OG0000000	*PyolGH55_1 PypeGH55_4*
	OG0000001	*PyolGH55_2 PypeGH55_3*
	OG0000002	*PyolGH55_5 PypeGH55_5*
	OG0000003	*PyolGH55_3 PypeGH55_2*
	OG0000004	*PyolGH55_4 PypeGH55_1*
GH71 family	OG0000000	*PyolGH71-1 PypeGH71-1*
	OG0000001	*PyolGH71-2 PypeGH71-2*

	OG0000002	*PyolGH71-3 PypeGH71-3*
GH19 family	OG0000000	*PagGH19-1 PcGH19-1 PcGH19-2 PiGH19-1 PiGH19-2 PrGH19-1 PveGH19-1 PveGH19-2 PveGH19-3 PveGH19-4 PyinGH19-2 PyinGH19-3 PyolGH19-12 PyolGH19-13 PyolGH19-3 PyolGH19-4 PyolGH19-5 PyolGH19-6 PyolGH19-7 PyolGH19-8 PypeGH19-5 PypeGH19-6*
	OG0000001	*PyinGH19-1 PyolGH19-11 PypeGH19-3*
	OG0000002	*PsGH19-1 PsGH19-2*
	OG0000003	*PyolGH19-1 PypeGH19-1*
	OG0000004	*PyolGH19-2 PypeGH19-2*
	OG0000005	*PyolGH19-9 PypeGH19-4*
	OG0000006	*PyolGH19-10*
GH46 family	OG0000000	*PyolGH46_1 PypeGH46_1*
	OG0000001	*PyolGH46_2 PypeGH46_2*
	OG0000002	*PyolGH46_3 PypeGH46_3*
GH76 family	OG0000000	*PyolGH76-3 PypeGH76-4*
	OG0000001	*PyolGH76-1 PypeGH76-2*
	OG0000002	*PyolGH76-2 PypeGH76-3*
	OG0000004	*PyolGH76-4*
	OG0000005	*PypeGH76-1*
GH43_26 family	OG0000000	*PyolGH43_26-1 PypeGH43_26-2 PypeGH43_26-4 PypeGH43_26-7 PypeGH43_26-8*
	OG0000001	*PypeGH43_26-1*
	OG0000002	*PypeGH43_26-3*
	OG0000003	*PypeGH43_26-5*
	OG0000004	*PypeGH43_26-6*
CBEL family	OG0000000	*PagCBEL-2 PiCBEL-1 PirCBEL-1 PirCBEL-2 PirCBEL-4 PiwCBEL-1 PiwCBEL-2 PiwCBEL-9 PrCBEL-1 PrCBEL-11 PrCBEL-12 PrCBEL-13 PrCBEL-2 PsCBEL-1 PsCBEL-2 PyinCBEL-1 PyolCBEL-2 PyolCBEL-4 PyolCBEL-5 PypeCBEL-2 PypeCBEL-3 PypeCBEL-4 PyuCBEL-5*
	OG0000001	*PcCBEL-2 PcCBEL-5 PiCBEL-4 PirCBEL-3 PiwCBEL-3 PiwCBEL-7 PrCBEL-14 PrCBEL-4 PsCBEL-3 PsCBEL-4 PsCBEL-5 PsCBEL-7 PugCBEL-2 PyuCBEL-1 PyuCBEL-2*
	OG0000002	*PagCBEL-1 PiCBEL-2 PiwCBEL-6 PiwCBEL-8 PrCBEL-7 PsCBEL-9 PveCBEL-2 PyolCBEL-1 PyolCBEL-3 PypeCBEL-1 PypeCBEL-5 PyuCBEL-6*
	OG0000003	*PrCBEL-10 PrCBEL-8 PrCBEL-9 PugCBEL-1 PyolCBEL-6 PypeCBEL-6 PyuCBEL-3 PyuCBEL-4*
	OG0000004	*PagCBEL-3 PiCBEL-3 PiwCBEL-4 PiwCBEL-5 PrCBEL-3 PsCBEL-10*
	OG0000005	*ParCBEL-1 PcCBEL-1 PcCBEL-3 PveCBEL-1 PagCBEL-4*
	OG0000006	*PcCBEL-4 PrCBEL-5 PrCBEL-6 PsCBEL-6 PveCBEL-3*
	OG0000007	*PcCBEL-6 PirCBEL-5 PsCBEL-8*
CesA family	OG0000000	*AaCesA-2.1 AaCesA-2.2 HpCesA-1 HpCesA-2 PcCesA-1 PcCesA-2 PiCesA-1 PiCesA-2 PrCesA-1 PrCesA-2 PsCesA-1 PsCesA-2 PyagCesA-1 PyagCesA-2 PyarCesA-1 PyarCesA-2 PygCesA-2 PyinCesA-1 PyinCesA-2 PyirCesA-1 PyirCesA-2 PyiwCesA-1*

		PyiwCesA-2 PyolCesA-1 PyolCesA-2 PypeCesA-2 PyuCesA-1 PyuCesA-2 PyuVarCesA-1 PyveCesA-1 SpCesA-2.1 SpCesA-2.2 SpCesA-2.3 PyveCesA-2
	OG0000001	*AaCesA-3.1 AaCesA-3.2 HpCesA-3 PcCesA-3 PiCesA-3 PsCesA-3 PyagCesA-3.1 PyagCesA-3.2 PyarCesA-3 PygCesA-3 PyinCesA-3.1 PyinCesA-3.2 PyirCesA-3 PyiwCesA-3 PyolCesA-3.1 PyolCesA-3.2 PypeCesA-3 PyuCesA-3 PyuVarCesA-3 PyveCesA-3 SpCesA-3.1 SpCesA-3.2*
	OG0000002	*AaCesA-4 PcCesA-4 PiCesA-4 PrCesA-4 PsCesA-4 PyagCesA-4 PyarCesA-4 PygCesA-4 PyinCesA-4 PyirCesA-4 PyiwCesA-4 PyolCesA-4 PypeCesA-4 PyuCesA-4 PyuVarCesA-4 PyveCesA-4 SpCesA-4*
